# Comprehensive profiling of the TRIpartite motif family to identify pivot genes in hepatocellular carcinoma

**DOI:** 10.1002/cam4.4552

**Published:** 2022-02-09

**Authors:** Lingyun Wu, Xin Yin, Kan Jiang, Jie Yin, Hao Yu, Lingling Yang, Chiyuan Ma, Senxiang Yan

**Affiliations:** ^1^ Department of Radiation Oncology, The First Affiliated Hospital Zhejiang University School of Medicine Hangzhou China; ^2^ Department of Gastroenterology the Second Affiliated Hospital of Nanchang University Nanchang China; ^3^ Department of Orthopaedic Surgery, The Second Affiliated Hospital Zhejiang University School of Medicine Hangzhou China

**Keywords:** hepatocellular carcinoma, immune microenvironment, TRIpartite motif (TRIM) family, tumor prognosis

## Abstract

**Introduction:**

TRIpartite motif (TRIM) proteins are important members of the Really Interesting New Gene‐finger‐containing E3 ubiquitin‐conjugating enzyme and are involved in the progression of hepatocellular carcinoma (HCC). However, the diverse expression patterns of TRIMs and their roles in prognosis and immune infiltrates in HCC have yet to be analyzed.

**Materials:**

Combined with previous research, we used an Oncomine database and the Human Protein Atlas to compare TRIM family genes' transcriptional levels between tumor samples and normal liver tissues, as verified by the Gene Expression Profiling Interactive Analysis database. We investigated the patient survival data of TRIMs from the Kaplan–Meier plotter database. Clinicopathologic characteristics associations and potential diagnostic and prognostic values were validated with clinical and expressional data collected from the cancer genome atlas.

**Results:**

We identified TRIM28, TRIM37, TRIM45, and TRIM59 as high‐priority members of the TRIMs family that modulates HCC. Low expression of TRIM28 was associated with shorter overall survival (OS) than high expression (log‐rank *p* = 0.009). The same trend was identified for TRIM37 (*p* = 0.001), TRIM45 (*p* = 0.013), and TRIM59 (*p* = 0.011). Multivariate analysis indicated that the level of TRIM37 was a significant independent prognostic factor for both OS (*p* = 0.043) and progression‐free interval (*p* = 0.044). We performed expression and mutation analysis and functional pathways and tumor immune infiltration analysis of the changes in TRIM factors.

**Conclusion:**

These data suggested that TRIM28, TRIM37, TRIM45, and TRIM59 could serve as efficient prognostic biomarkers and therapeutic targets in HCC.

## Graphical abstract

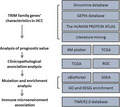

## 1 INTRODUCTION

Hepatocellular carcinoma (HCC) is the sixth most common cancer in the world and was the third leading cause of cancer‐related death in 2020.^1^ The number of newly diagnosed HCC cases is increasing worldwide. This is a common aggressive malignancy in developing countries. Liver resection and transplantation are treatment options in selected patients, but surgery is often followed by HCC recurrence.^2^ The 5‐year rate of HCC recurrence is as high as 70%.^3^

The majority of patients are not eligible for this radical curative therapy, however, which has a median survival of <1 year.^4^ While multitargeted kinase inhibitors received FDA approval for HCC treatment, there are still no reliable biomarkers for accurate patient’ prognosis due to tumor heterogeneity. There is an urgent need for novel molecular markers to effectively enhance prognosis.

The TRIpartite motif (TRIM) family of proteins, previously designated as RBCC for the presence of a Really Interesting New Gene (RING)‐finger domain, zinc‐finger domains named B‐box motifs, and the associated Coiled‐Coil region were first identified through a functional genomic approach as 37 proteins sharing common functions and cellular compartments.^5^, ^6^ Similar proteins have been discovered, including more than 80 TRIM protein genes in humans. Among these, only eight TRIM proteins are RING‐less.^7^ The RING‐finger domain containing‐TRIM protein family and the homologous to E6‐AP COOH terminus (HECT) family constitute the two parts of the E3 ubiquitin ligases, which catalyzes the ubiquitin conjugation with E1 ubiquitin‐activating enzyme and E2 ubiquitin‐conjugating enzymes.^8^

Although they differ in the C‐terminus and overall domain structure, TRIM proteins participate in diverse cellular mechanisms and biologic processes, including gene expression, cell cycle progression, apoptosis, signal transduction, developmental processes, and carcinogenesis.^9^ Evidence has demonstrated that a variety of TRIM protein family members play vital roles in promoting proliferation, migration, and invasion of HCC cells. A close association between TRIM proteins and poor survival has been noted.^10^, ^11^, ^12^, ^13^ Some TRIM proteins are of value in HCC and the explicit roles of TRIM family gene’ expression on patient’ diagnosis, disease progression, and immune regulation in a tumor microenvironment must be clarified.

No systematical analysis has been performed to identify the role of TRIMs in HCC. Here we performed a comprehensive bioinformatics analysis with genome‐sequencing technology and explored various public databases, to determine whether TRIM proteins have potential as therapeutic targets and prognostic biomarkers in HCC. Our study suggests that TRIM28, TRIM37, TRIM45, and TRIM59 may be useful to stratify the prognosis in HCC patients. These four TRIM family members hold promise for HCC diagnosis and treatment.

## 2 METHOD

### 2.1 Oncomine analysis

A meta‐analysis of TRIMs in cancer expression was performed using an online cancer microarray database Oncomine (www.oncomine.org).^14^ The fold change and *p* value cutoffs were defined as 2 and 0.01, respectively. The heatmap figures were generated for patient annotated gene expression.

### 2.2 Protein atlas analysis

The Human Protein Atlas (HPA) provides qualitative antibody‐based annotation of protein expression levels across 76 different cell types in 44 human tissue categories. Samples with immunohistochemistry scoring intensity in HCC tissues were included.

### 2.3 GEPIA data set

The online Gene Expression Profiling Interactive Analysis (GEPIA) platform (http://gepia.cancer‐pku.cn/) is an analysis tool containing the RNA sequence expression data of 9736 tumors and 8587 normal tissue samples.^15^ In this study, we conducted a differential expression analysis for the mRNA of HCC and normal liver tissues as well as clinicopathologic analyses of TRIMs with GEPIA. The “Multiple Gene Comparison” module of the “LIHC” data set was used to conduct multiple gene comparison analysis of TRIMs. The *p* value was assessed by Student's *t* test and the cutoff was 0.05.

### 2.4 The Kaplan–Meier plotter

The prognostic values of TRIMs mRNA expression were evaluated using the Kaplan–Meier plotter database (www.kmplot.com), which contains gene expression data and survival information for HCC patients. To analyze four indicators, the overall survival (OS), progress‐free survival (PFS), relapse‐free survival (RFS), and disease‐specific survival (DSS) of patients with HCC, clinical samples were split according to median expression and estimated by a Kaplan–Meier survival plot. Forest plots were used to present the Cox regression models.

### 2.5 cBioPortal

The cBioPortal (http://cbioportal.org) provides researchers to explore and analyze multidimensional genomics data.^16^ According to the TCGA database, genomic profiles were obtained from cBioPortal with the threshold of ±2.0.

### 2.6 Functional enrichment and TRIMs‐related pathway analysis

The KEGG pathway and GO term analysis have been previously described.^17^ To better analyze and visualize the functional profiles of TRIMs, we used the R package clusterProfiler (3.8.0). Next, to prepare for the gene set enrichment analysis (GSEA), the ratio between the TRIMs‐high and ‐low groups in HCC was calculated. The false discovery rate (FDR) *q* value of <0.25, the adjusted *p* value of <0.05, and the absolute normalized enrichment score (NES) of >1 were used to select the most significantly enriched signaling pathways.

### 2.7 Coexpression network analysis

The correlations of the four TRIM members with each other were obtained by analyzing mRNA expressions (RNA‐sequencing [RNA‐seq] version (v.)2 RSEM) from the XIANTAO platform (www.xiantao.love) from the TCGA project according to the online instructions.

### 2.8 TIMER 2.0

The TIMER 2.0 database (http://timer.cistrome.org/) is a comprehensive tool that quantifies tumor‐infiltrating immune cells and provides analysis of immune infiltration with cancer molecular profiles.^18^ The website is enabled with an intuitive interface, convenient visualization of the analysis results, and a flexible selection of particular methodologies. In the present study, the “gene module” was used to estimate the interrelationship between TRIM expression and immune cell infiltration. The “survival module” was used to evaluate the interrelationship between the assessment of clinical outcomes and the infiltration of immune cells and TRIM levels. We adopted the Wilcoxon‐rank sum test and Spearman correlation.

### 2.9 Statistical analysis

Data and figures were mainly processed using SPSS 23.0 and the GraphPad Prism 8.0 software. Values are displayed as the mean ± *SD*. The association between TRIM expression and clinicopathologic features in HCC patients was analyzed with the Wilcoxon signed‐rank sum test and Spearman's or Pearson's test. Survival analyses were conducted with the log‐rank test and the Cox proportional hazard regression model. The cutoff value of the TRIM levels was dependent on the median value. A probability value <0.05 was considered a statistically significant result. Significance was defined at ***p < 0.001, **p < 0.01, and *p < 0.05.

## 3 RESULTS

### 3.1 Expression levels of TRIM family genes and comparison between HCC and normal liver tissues

**FIGURE 1:**
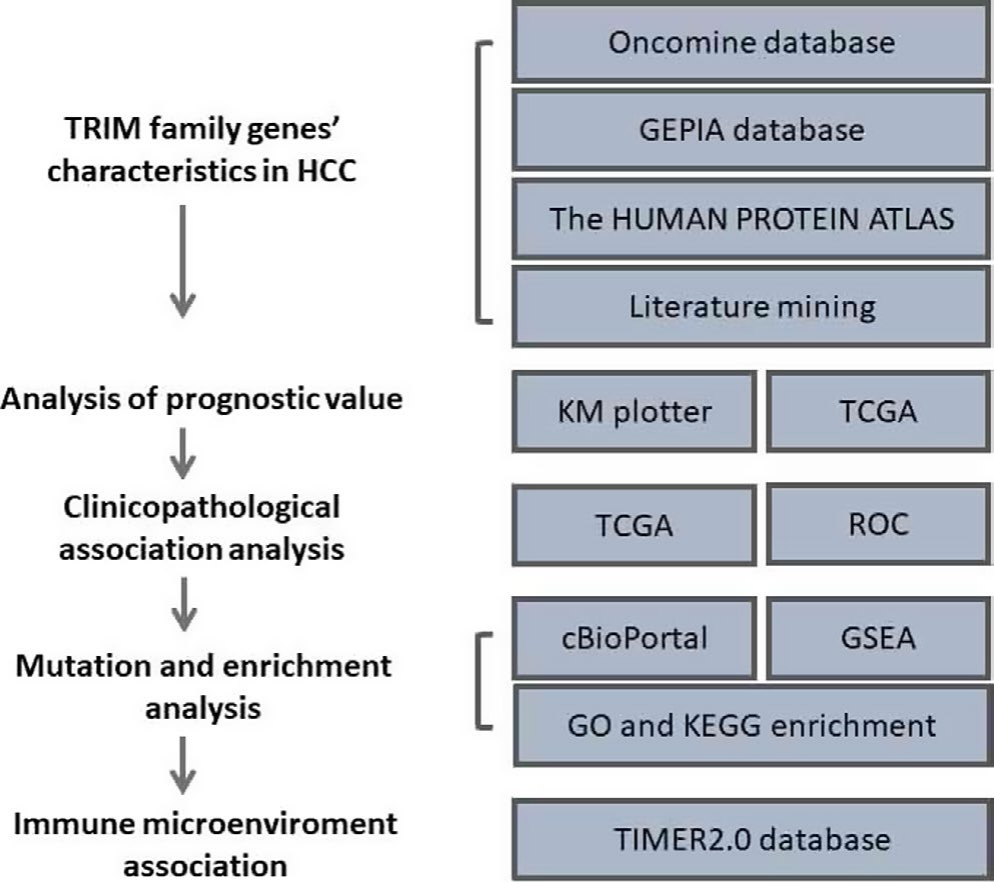
Flow chart of data collection and analysis. TCGA, the cancer genome atlas; ROC, receiver operator characteristic; GSEA, gene set enrichment analysis; GO, gene ontology; KEGG, kyoto encyclopedia of genes and genomes

**FIGURE 2:**
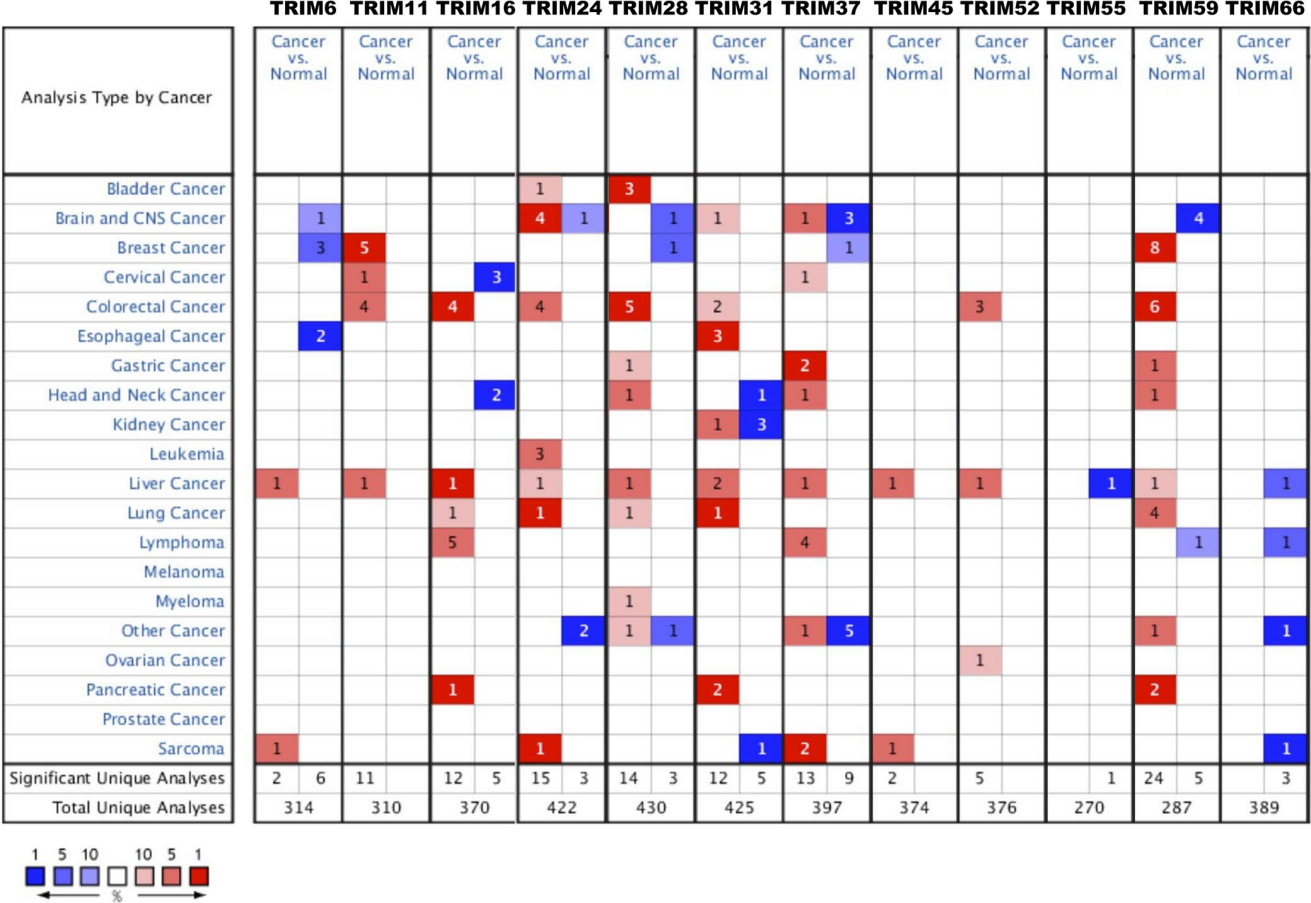
Transcription levels of TRIM family members in different types of cancers (Oncomine). The figure shows the numbers of datasets with statistically significant upregulation (red) or downregulated expression (blue) of TRIMs

Figure 1 shows a flowchart of the screening process for the targeted TRIM family genes correlated with HCC. A total of 76 TRIM factors were manually retrieved using the Oncomine database. We first compared the transcriptional levels of TRIMs in HCC with those in normal liver tissues (Table 1; Figure 2). Based on Oncomine, the mRNA expression levels of TRIM6, TRIM11, TRIM16, TRIM24, TRIM28, TRIM31, TRIM37, TRIM45, TRIM52, and TRIM59 in HCC tissues were significantly elevated in HCC versus normal liver tissue, and TRIM55 was remarkably downregulated in normal liver tissues.

**TABLE 1:** TRIMs expression in mRNA level between hepatocellular carcinoma and normal liver tissues (Oncomine)

	Type of liver disease	Fold change	*p* value	*t* test	Source and/or references
TRIM6	Hepatocellular carcinoma	2.178	7.15E‐6	4.906	Wurmbach Liver Statistics^19^
TRIM11	Hepatocellular carcinoma	2.230	7.26E‐7	7.783	Wurmbach Liver Statistics^19^
	Hepatocellular carcinoma	1.702	1.00E‐18	9.882	Chen Liver Statistics^20^
	Focal Nodular Hyperplasia	1.614	0.026	2.924	Chen Liver Statistics^20^
TRIM16	Hepatocellular carcinoma	5.726	7.82E‐9	7.072	Wurmbach Liver Statistics^19^
	Hepatocellular carcinoma	1.779	4.38E‐7	5.677	Mas Liver Statistics^21^
	Cirrhosis	1.029	0.262	0.643	Mas Liver Statistics^21^
	Hepatocellular carcinoma	2.601	6.96E‐28	12.330	Roessler Liver Statistics^22^
TRIM24	Hepatocellular carcinoma	2.046	4.84E‐44	15.816	Roessler Liver Statistics^22^
	Hepatocellular carcinoma	1.957	8.45E‐16	8.744	Chen Liver Statistics^20^
	Focal Nodular Hyperplasia	1.246	0.167	1.123	Chen Liver Statistics^20^
	Hepatocellular carcinoma	1.868	1.00E‐5	4.898	Wurmbach Liver Statistics^19^
	Hepatocellular carcinoma	1.231	4.82E‐4	3.489	Mas Liver Statistics^21^
	Cirrhosis	1.029	0.284	0.575	Mas Liver Statistics^21^
	Hepatocellular carcinoma	1.047	1.29E‐6	4.958	Guichard Liver Statistics^23^
	Hepatocellular carcinoma	1.062	6.80E‐4	3.299	TCGA
TRIM28	Hepatocellular carcinoma	1.802	2.53E‐41	15.210	Roessler Liver Statistics^22^
	Hepatocellular carcinoma	1.529	7.36E‐10	6.388	Chen Liver Statistics^20^
	Hepatocellular carcinoma	1.712	2.53E‐4	4.115	Wurmbach Liver Statistics^19^
TRIM31	Hepatocellular carcinoma	3.010	1.92E‐6	5.349	Wurmbach Liver Statistics^19^
	Hepatocellular carcinoma	1.286	4.27E‐7	5.599	Mas Liver Statistics^21^
	Hepatocellular carcinoma	1.369	3.15E‐8	5.660	Chen Liver Statistics^20^
	Hepatocellular carcinoma	1.058	3.35E‐9	6.307	Guichard Liver Statistics^23^
	Hepatocellular carcinoma	1.114	8.06E‐9	6.162	TCGA
	Hepatocellular carcinoma	1.397	4.03E‐17	8.808	Roessler Liver Statistics^22^
TRIM37	Hepatocellular carcinoma	2.183	3.76E‐60	19.299	Roessler Liver Statistics^22^
	Hepatocellular carcinoma	1.098	2.02E‐8	5.966	TCGA
	Hepatocellular carcinoma	1.039	6.63E‐6	4.530	Guichard Liver Statistics^23^
	Hepatocellular carcinoma	1.358	0.003	3.208	Wurmbach Liver Statistics^19^
TRIM45	Hepatocellular carcinoma	2.292	1.33E‐10	6.785	Chen Liver Statistics^20^
	Hepatocellular carcinoma	1.172	6.03E‐17	8.710	Roessler Liver Statistics^22^
TRIM52	Hepatocellular carcinoma	2.319	1.76E‐6	6.163	Wurmbach Liver Statistics^19^
	Hepatocellular carcinoma	1.148	0.004	2.783	Mas Liver Statistics^21^
	Hepatocellular carcinoma	1.303	2.42E‐5	4.174	Chen Liver Statistics^20^
	Hepatocellular carcinoma	1.338	4.17E‐18	9.027	Roessler Liver Statistics^22^
TRIM55	Hepatocellular carcinoma	1.105	5.89E‐11	7.192	Guichard Liver Statistics^23^
	Hepatocellular carcinoma	1.191	5.09E‐11	7.258	TCGA
TRIM59	Hepatocellular carcinoma	2.250	1.15E‐7	5.438	Chen Liver Statistics^20^
	Hepatocellular carcinoma	1.602	8.08E‐5	4.137	Wurmbach Liver Statistics^19^
	Hepatocellular carcinoma	1.027	0.009	2.426	TCGA

Abbreviation: TRIM, TRIpartite motif.

Specifically, the mRNA level of TRIM6 was overexpressed in patients with HCC, with a fold change of 2.178 in Wurmbach's data set (Table 1). Meanwhile, Wurmbach et al^19^ also showed that TRIM11 was increased in those with HCC (fold change = 2.230 and *p* value = 7.26E‐7). Chen et al^20^ found a statistically significant upregulation of TRIM11 in HCC (fold change = 1.702 and *p* = 1.00E‐18) as well as in focal nodular hyperplasia (fold change = 1.614 and *p* = 0.026). Three data sets suggested that TRIM16 expression was elevated in HCC^19^, ^21^, ^22^ compared with normal tissues (with the fold change of 5.726, 1.779, and 2.601, respectively).

No statistical significance was reported between cirrhosis and liver samples.^21^ A total of five data sets and TCGA liver statistical data reported the overexpression of TRIM24 in HCC. In Roessler's data set, TRIM24 was significantly elevated in HCC with a fold change of 2.046.^22^ Chen et al also indicated that the level of TRIM24 (*p* = 8.45E‐16) in HCC was remarkably elevated with a fold change of 1.957, yet no statistical significance was found in focal nodular hyperplasia compared with normal liver tissues (*p* = 0.167).^20^ Similarly, the results of Wurmbach,^19^ Mas,^21^ and Guichard^23^ all implied that TRIM24 increased significantly in HCC tumor tissues compared with normal samples, which is consistent with the results of TCGA liver statistics. The transcriptional levels of TRIM28 in HCC were remarkably higher than in normal liver tissues in the Roessler (fold change = 1.802 and *p* = 2.53E‐41), Chen (fold change = 1.529 and *p* = 7.36E‐10), and Wurmbach (fold change = 1.712 and *p* = 2.53E‐4) data sets.^19^, ^20^, ^22^

The fold change of TRIM31 expression in HCC was 3.010 (*p* = 1.92E‐6), 1.286 (*p* = 4.27E‐7), 1.369 (*p* = 3.15E‐8), 1.058 (*p* = 3.35E‐9), and 1.397 (*p* = 4.03E‐17) in the data sets of Wurmbach,^19^ Mas,^21^ Chen,^20^ Guichard,^23^ and Roessler,^22^ respectively. In TCGA (Table 1), TRIM31 was also overexpressed in HCC (fold change = 1.114 and *p* = 8.06E‐9) compared with normal liver tissues. TCGA liver statistics reported a significant upregulation of TRIM37 in HCC (fold change = 1.098 and *p* = 2.02E‐8). Similarly, the results of Roessler,^22^ Guichard,^23^ and Wurmbach^19^ all suggested that TRIM37 increased significantly in HCC tumors compared with normal liver samples (with fold change of 2.183, 1.039, and 1.358, respectively). Chen et al^20^ showed upregulation of TRIM45 in HCC (fold change = 2.292 and *p* = 1.33E‐10). The same observation was found in the Roessler data set^22^ (fold change = 1.172 and *p* = 6.03E‐17).

The results of Wurmbach,^19^ Mas,^21^ Chen,^20^ and Roessler^22^ all suggest that TRIM52 is significantly upregulated in HCC with a fold change of 2.319 (*p* = 1.76E‐6), 1.148 (*p* = 0.004), 1.303 (*p* = 2.42E‐5), and 1.338 (*p* = 4.17E‐18), respectively. TCGA liver statistics suggest that TRIM55 is overexpressed in HCC tissues (fold change = 1.191 and *p* = 5.09E‐11), yet Wurmbach's data set reported a significant downregulation of TRIM55 in normal liver tissues compared with HCC samples. Moreover, per TCGA liver statistics, both Chen et al^20^ and Wurmbach et al^19^ showed that increased TRIM59 was found in HCC tissues compared with normal samples (fold change = 2.250 and *p* = 1.15E‐7, fold change = 1.602 and *p* = 8.08E‐5, respectively).

### 3.2 Validation of aberrant expression of TRIMs in HCC

**FIGURE 3:**
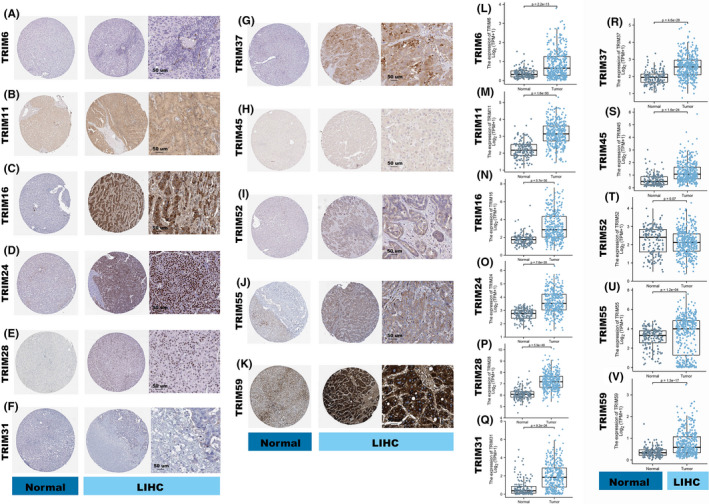
TRIMs expression in HCC and normal liver tissue. (A–K) Representative immunohistochemistry results of the selected TRIM members between normal (left) and tumor (right) tissues based on the human protein atlas. Original magnification ×400. (L–V) Comparison of TRIMs gene expression between normal liver samples and tumor tissues. LIHC, Liver hepatocellular carcinoma

To validate TRIM family expressions at the protein level, we assessed immunohistochemical results obtained from the HPA database and compared the outcomes from the TCGA and GTEx databases. Representative images are shown in Figure 3.

TRIM6 protein levels showed negative staining and intensity based on the HPA, yet a significantly higher level of TRIM6 was detected at the transcriptional level (*p* = 2.2E‐13) (Figure 3A,L). TRIM11 demonstrated low staining and weak intensity in normal liver samples and as medium staining and moderate intensity in HCC tissue, which was verified in the TCGA and GTEx databases (*p* = 1.6E‐50) (Figure 3B,M). Significant upregulation of TRIM16, TRIM24, and TRIM28 in HCC tissue was detected with high staining and strong intensity by the HPA, consistent with assessment at the transcriptional level in the TCGA and GTEx databases (*p* = 5.7E‐30, *p* = 7.6E‐35, and *p* = 5.9E‐49, respectively) (Figure 3C–E,N–P).

Negative staining and intensity were detected from TRIM31 in both HCC and normal liver tissue, while significantly higher expression was found in the TCGA and GTEx databases (*p* = 9.2E‐26) (Figure 3F,Q). Protein and mRNA levels of TRIM37, TRIM52, TRIM55, and TRIM59 were increased in HCC tissues compared with normal liver samples by immunohistochemical experiments (*p* = 4.6E‐28, *p* = 0.07, *p* = 1.2E‐04 and *p* = 1.3E‐17, respectively) (Figure 3G,I–K,R,T–V). TRIM45 was detected with no significant difference based on the HPA, but with a higher expression in the HCC tissues than in the normal samples at the transcriptional level (*p* = 1.6E‐24) (Figure 3H,S).

### 3.3 Influence of TRIM family gene mRNA expressions on patients' survival

**FIGURE 4:**
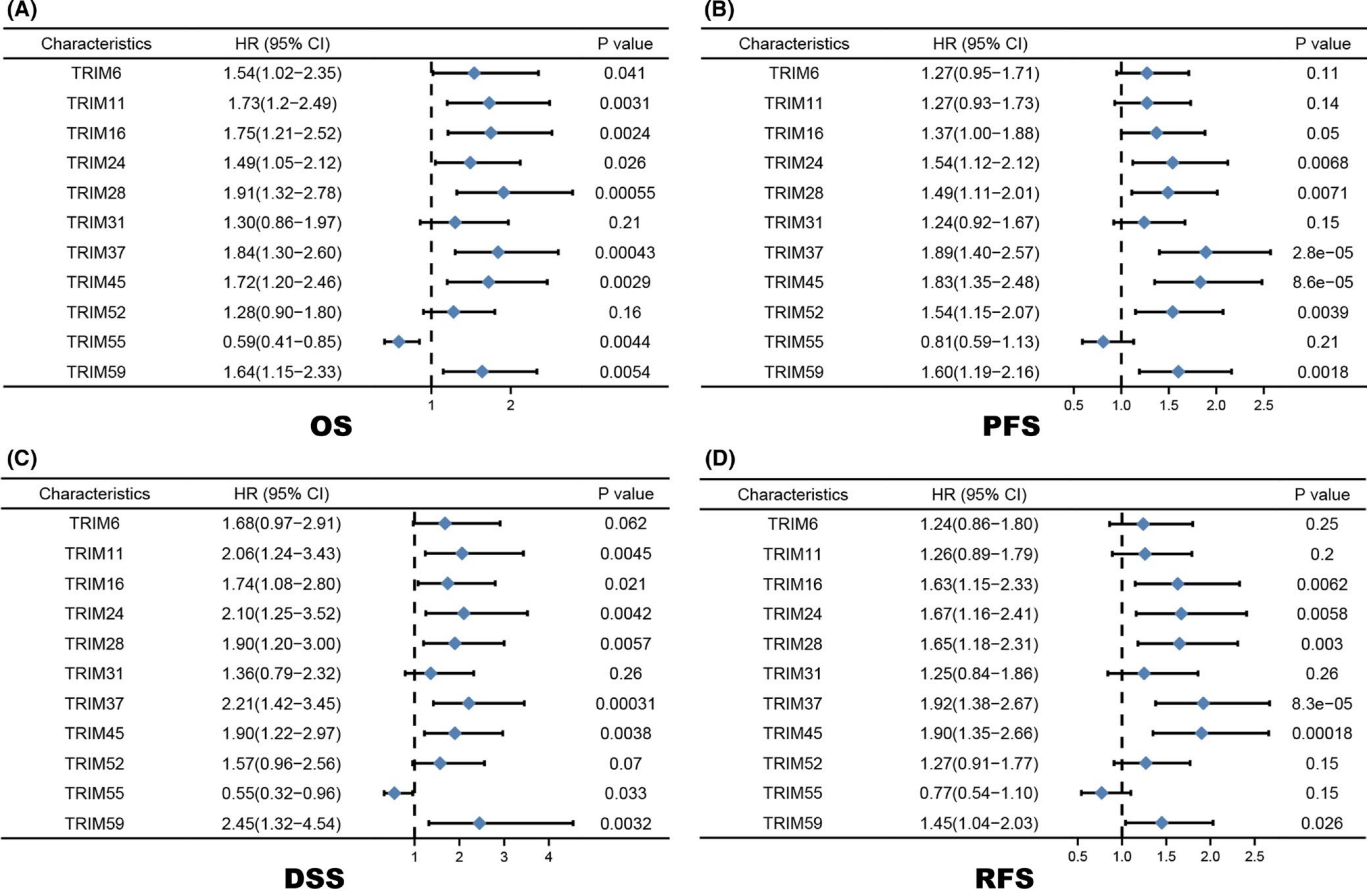
Forest plots were used to evaluate the association between TRIMs expression and (A) OS, (B) PFS, (C) DSS and (D) RFS of HCC patients

The association between TRIM family expressions and patients' survival was analyzed with the Kaplan–Meier plotter online database. The survival curves of OS, PFS, RFS, and DSS were plotted by GraphPad Prism (Figure 4).

Briefly, the Kaplan–Meier survival curve and log‐rank test analyses suggested that the increased TRIM24, TRIM28, TRIM37, TRIM45, and TRIM59 mRNA levels were significantly correlated with poor OS (*p* = 0.026, 0.00055, 0.00043, 0.0029, and 0.0054, respectively), PFS (*p* = 0.0068, 0.0071, 2.8E‐5, 8.6E‐5, and 0.0018, respectively), DSS (*p* = 0.0042, 0.0057, 0.00031, 0.0038, and 0.0032, respectively), and RFS (*p* = 0.0058, 0.003, 8.3E‐5, 0.00018, and 0.026, respectively) of all the patients with HCC. Similarly, upregulated levels of TRIM16 were associated with worse OS (*p* = 0.0024), DSS (*p* = 0.021), and RFS (*p* = 0.0062). We predicted that the patients with liver cancer with high TRIM11 and TRIM55 mRNA expression would have high OS (*p* = 0.0031 and 0.0044, respectively) and DSS (*p* = 0.0045 and 0.033, respectively). Moreover, the OS rate was significantly higher in TRIM6‐low HCC patients (*p* = 0.041), whereas the PFS rate was higher in the TRIM52‐low group (*p* = 0.0039). However, there was no significant difference detected in survival analyses of TRIM31.

**FIGURE 5:**
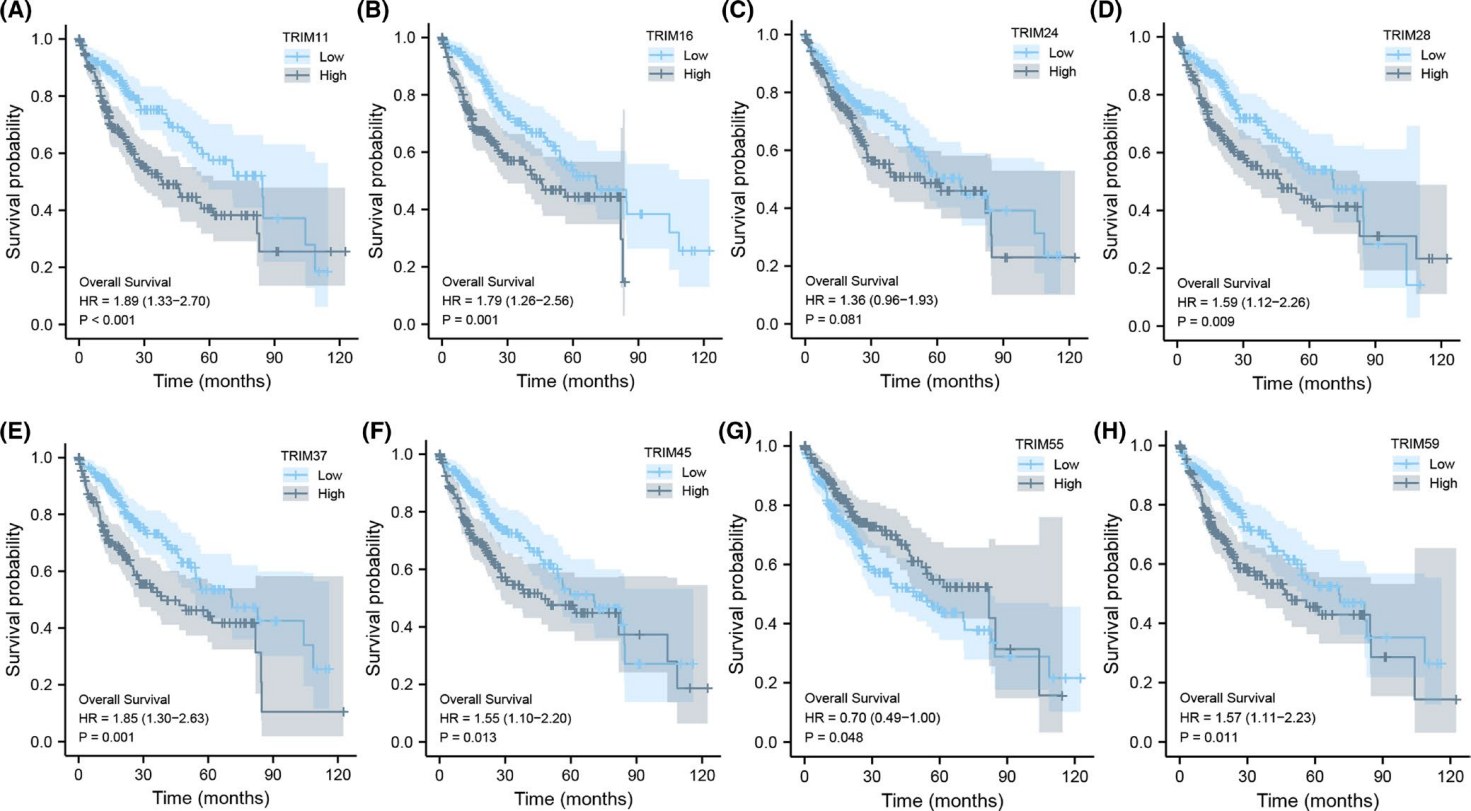
The prognostic value of different expressed TRIMs in HCC patients in the OS curve (TCGA). The OS curve of (A) TRIM11, (B) TRIM16, (C) TRIM24, (D) TRIM28, (E) TRIM37, (F) TRIM45, (G) TRIM55 and (H) TRIM59 in HCC

Next, to further verify the potential roles of TRIMs in predicting HCC survival, we performed a survival association analysis with the above‐mentioned LIHC projects collected in the TCGA database. In addition to TRIM24 (*p* = 0.081), HCC patients with lower transcriptional levels of TRIM11 (*p* < 0.001), TRIM16 (*p* = 0.001), TRIM28 (*p* = 0.009), TRIM37 (*p* = 0.001), TRIM45 (*p* = 0.013), and TRIM59 (*p* = 0.011) as well as higher levels of TRIM55 (*p* = 0.048) were significantly associated with longer OS (Figure 5).

We also examined lower expressions of TRIM24 (*p* = 0.014), TRIM28 (*p* = 0.002), TRIM37 (*p* = 0.025), TRIM45 (*p* = 0.001), and TRIM59 (*p* = 0.006) and higher expression of TRIM55 (*p* = 0.063) in predicting DSS in HCC patients (Figure S1). Collectively, the data suggest that TRIM28, TRIM37, TRIM45, and TRIM59 could be potential HCC. We selected these four members of the TRIMs family for further research.

### 3.4 Verification of TRIMs with prognostic signature in HCC

A univariate logistic regression analysis showed that high expression of TRIM28, TRIM37, TRIM45, and TRIM59 were associated with a worse OS (HR: 1.627; 95% CI: 1.149–2.304, *p* = 0.006; HR: 1.795; 95% CI: 1.264–2.549, *p* = 0.001; HR: 1.511; 95% CI: 1.068–2.136, *p* = 0.020; HR: 1.568; 95% CI: 1.106–2.223, *p* = 0.012) (Table 2). Moreover, TRIM37 and TRIM59 were negatively associated with DSS (HR: 1.838; 95% CI: 1.173–2.880, *p* = 0.008; HR: 1.811; 95% CI: 1.152–2.847, *p* = 0.010). TRIM28, TRIM37, TRIM45, and TRIM59 were associated with a worse progression‐free interval (PFI) (HR: 1.508; 95% CI: 1.127–2.018, *p* = 0.006; HR: 1.666; 95% CI: 1.244–2.230, *p* < 0.001; HR: 1.644; 95% CI: 1.228–2.202, *p* < 0.001; HR: 1.556; 95% CI: 1.161–2.085, *p* = 0.003) (Tables S1 and S2). To further explore potential clinical biomarkers associated with the survival of HCC patients, a multivariate Cox regression analysis was used to assess gender, age, TNM stage, pathologic stage, Child‐Pugh grade, AFP, albumin levels, and prothrombin time. Overexpressed TRIM37 was an independent prognostic factor associated with poor OS (HR: 1.663; CI: 1.016–2.720; *p* = 0.043) and PFI (HR: 1.511; CI: 1.011–2.257; *p* = 0.044). However, the expression levels of TRIM28, TRIM45, and TRIM59 showed no association with poor OS, DSS, or PFI in patients with HCC (Tables S1 and S2).

**TABLE 2:** Univariate regression and multivariate cox regression analyses for overall survival in patients with hepatocellular carcinoma

Characteristics	Univariate analysis		Multivariate analysis	
	Hazard ratio (95% CI)	*p* value	Hazard ratio (95% CI)	*p* value
Gender (M/F)	1.261 (0.885–1.796)	0.200		
Age (≤60/>60)	1.205 (0.850–1.708)	0.295		
T stage (1/2/3/4)	3.727 (1.936–7.174)	**<0.001**	3.062 (1.124–8.341)	**0.029**
N stage (0/1)	2.029 (0.497–8.281)	0.324		
M stage (0/1)	4.077 (1.281–12.973)	**0.017**		
Pathologic.stage (I/II/III/IV)	3.770 (1.193–11.916)	**0.024**		
Child‐Pugh.grade (A/B/C)	1.643 (0.811–3.330)	0.168		
Histologic.grade (G1/2/3/4)	1.470 (0.598–3.609)	0.401		
Adjacent hepatic tissue inflammation (0/1)0	1.194 (0.734–1.942)	0.475		
AFP (ng/ml) (≤400/>400)	1.075 (0.658–1.759)	0.772		
Albumin (g/dl) (<3.5/≥3.5)	0.897 (0.549–1.464)	0.662		
Prothrombin time (≤4/>4)	1.335 (0.881–2.023)	0.174		
TRIM28 (low/high)	1.627 (1.149–2.304)	**0.006**		
TRIM37 (low/high)	1.795 (1.264–2.549)	**0.001**	1.663 (1.016–2.720)	**0.043**
TRIM45 (low/high)	1.511 (1.068–2.136)	**0.020**		
TRIM59 (low/high)	1.568 (1.106–2.223)	**0.012**		

Abbreviation: TRIM, TRIpartite motif.

All the statistically significant numbers are bold.

### 3.5 Correlations between TRIMs expression and clinicopathologic factors

**FIGURE 6:**
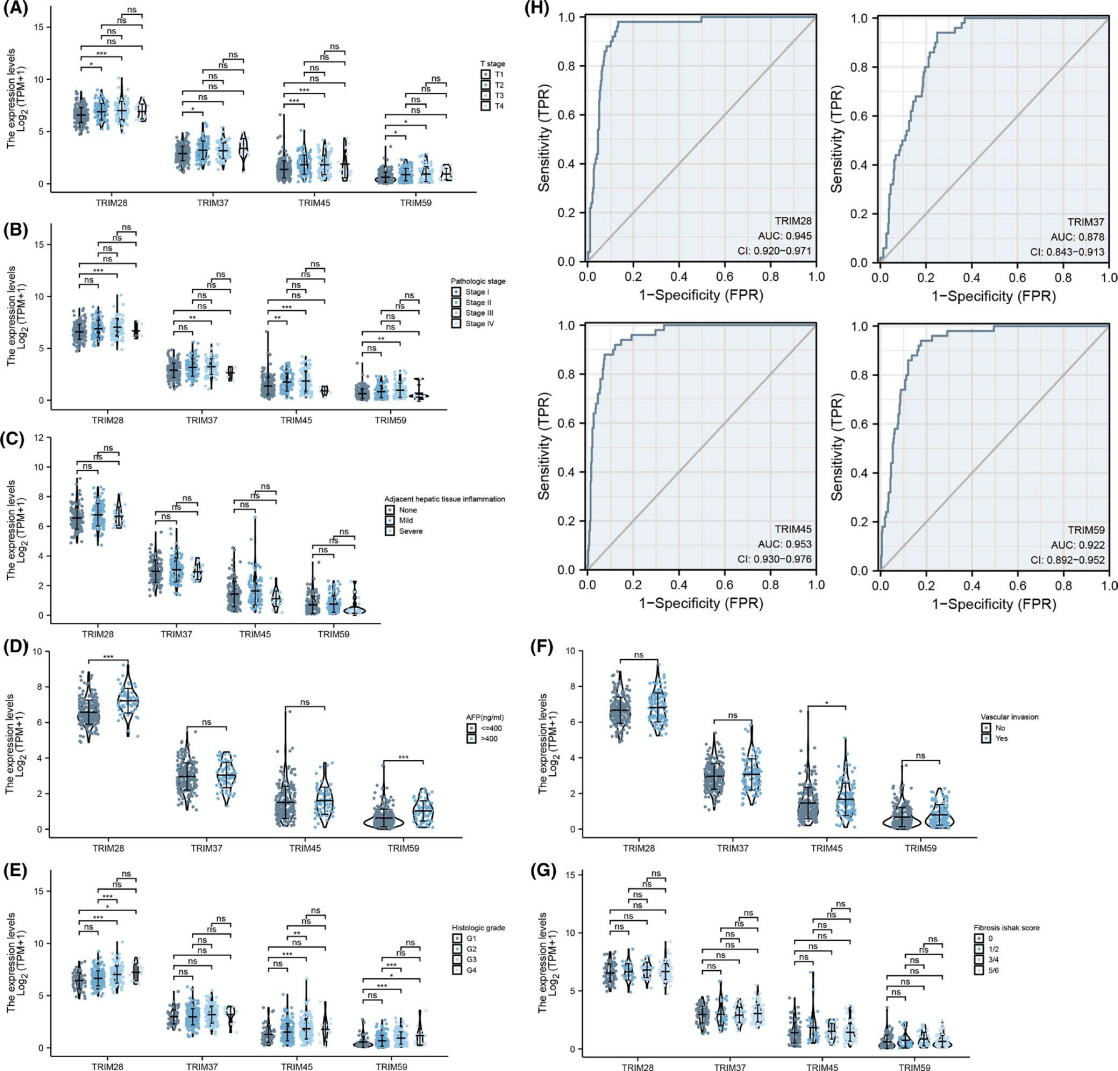
Association the expression level of TRIM28, TRIM37, TRIM45 and TRIM59 with clinicopathological characteristics of HCC patients, including (A) T stage, (B) pathologic stage, (C) adjacent hepatic tissue inflammation, (D)AFP level, (E)histologic grade, (F)vascular invasion and (G)Fibrosis Ishak score were visualized with violin plots. (H) ROC curve analysis and AUC analysis implemented to test the value of TRIMs to identify HCC tissues were also created. AUC, area‐under‐the‐curve

To definitively explore the role of TRIMs, a total of 424 HCC and matched normal liver tissues with TRIMs expression data as well as the patients' clinicopathologic characteristics were obtained from TCGA (Figure 6; Figure S2; Table S3). The cohort study consists of 122 women and 255 men of an average age of 59.45 (range 16–90) years. As presented in Figure 6A,B and D‐E, upregulated expression of TRIM28 is positively correlated with T stage (T1 vs. T2, *p* = 0.017; T1 vs. T3, *p* < 0.001), pathologic stage (stage I vs. stage III, *p* < 0.001), AFP level (≤400 vs. >400, *p* = 0.000), and histologic grade (G1 vs. G3, *p* < 0.001; G1 vs. G4, *p* = 0.021; G2 vs. G3, *p* < 0.001). Overexpressed TRIM37 is significantly correlated with T stage (T1 vs. T2, *p* = 0.035) and pathologic stage (stage I vs. stage III, *p* = 0.003) (Figure 6A,B). Increased level of TRIM45 in HCC is positively associated with T stage (T1 vs. T2, *p* < 0.001; T1 vs. T3, *p* = 0.001), pathologic stage (stage I vs. stage III, *p* = 0.003), histologic grade (G1 vs. G3, *p* < 0.001; G2 vs. G3, *p* = 0.009), and vascular invasion *(p*) (Figure 6A,B,E,F). In addition, higher expression of TRIM59 is significantly correlated with T stage (T1 vs. T2, *p* = 0.011; T1 vs. T3, *p* = 0.027), pathologic stage (stage I vs. stage III, *p* < 0.001), AFP level (≤400 vs. >400, *p* < 0.001), and histologic grade (G1 vs. G3, *p* < 0.001; G1 vs. G4, *p* = 0.019; G2 vs. G3, *p* < 0.001) (Figure 6A,B,D,E). However, ther was no significant difference of TRIMs in adjacent hepatic tissue inflammation and Fibrosis Ishak score (Figure 6C,G).

Receiver operator characteristic (ROC) curves were produced to analyze the diagnostic efficacy of TRIMs between HCC and normal liver tissues using the pROC package. As shown in Figure 6H, the area under the curve of TRIM28, TRIM37, TRIM45, and TRIM59 is 0.945 (95% CI: 0.920–0.971), 0.878 (95% CI: 0.843–0.913), 0.953 (95% CI: 0.930–0.976), and 0.922 (95% CI: 0.892–0.952) strongly suggesting that all of the selected TRIM family members could effectively differentiate HCC patients.

### 3.6 Genetic alteration, coexpression, and pathway and interaction analyses of TRIMs in patients with HCC

**FIGURE 7:**
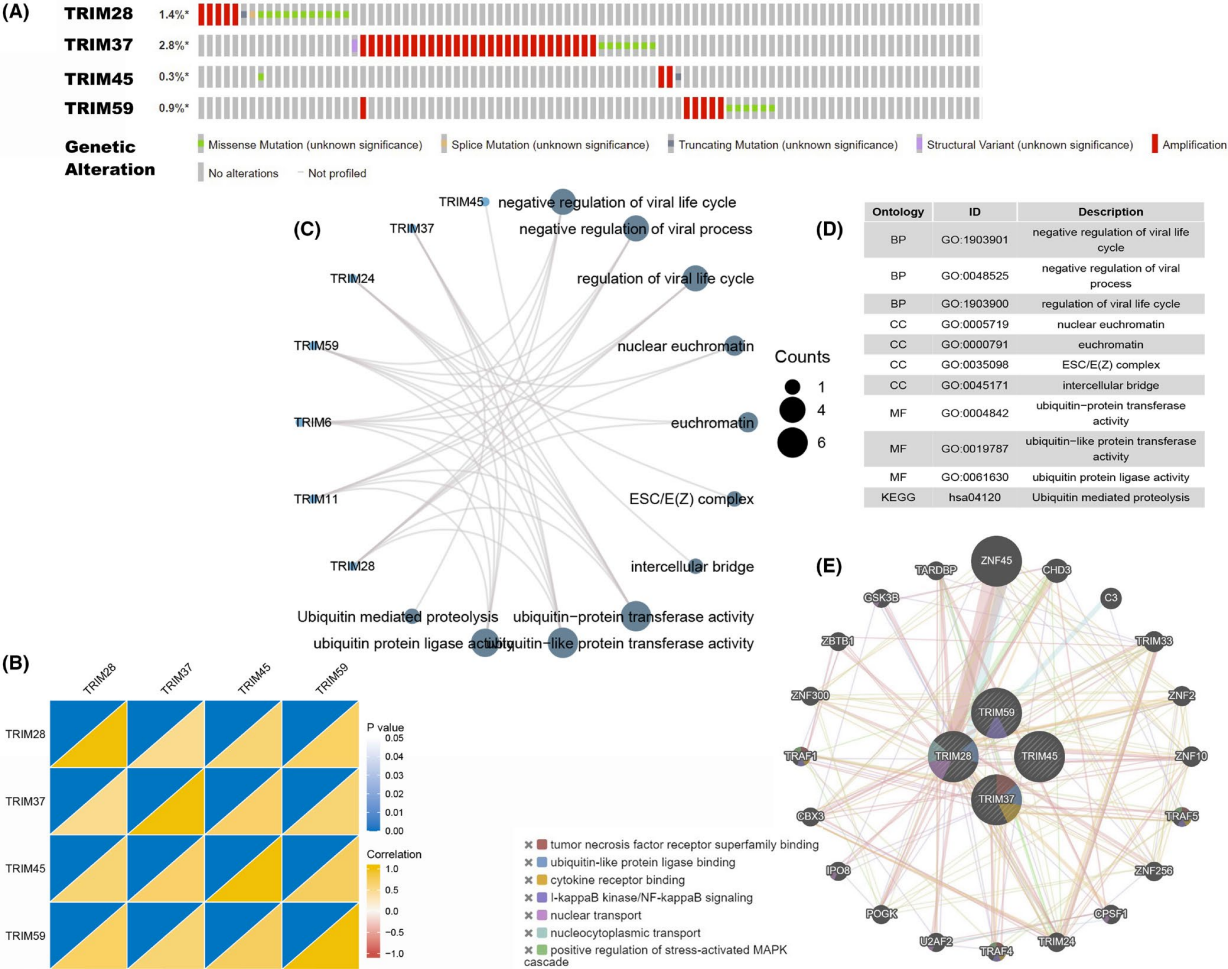
Genetic alteration, coexpression, interaction and GO function and KEGG pathway analyses of TRIMs in patients with HCC. (A) Summary of TRIMs gene mutation analysis in HCC (cBioPortal). (B) Correlation heat map of different expressed TRIMs in HCC (TCGA). (C–D) Gene functional enrichment of TRIM28, TRIM37, TRIM45 and TRIM59 in HCC. Top 10 biological processes, molecular functions and signaling pathways were collected in the table. (E) The network for TRIMs and the 20 most functionally similar genes as well as their pathways using genomics and proteomics data

We used the cBioPortal online tool for HCC to comprehensively analyze the selected family members of TRIM alterations. As shown in Figure 7A, TRIM28, TRIM37, TRIM45, and TRIM59 were altered in 1.4%, 2.8%, 0.3%, and 0.9% of the queried HCC samples, respectively. Amplification and missense mutations were the most common changes. Interestingly, the expression levels of TRIM28, TRIM37, TRIM45, and TRIM59 were significantly and positively correlated (Figure 7B).

To assess the role of the selected TRIMs in biologic processes, GO function and KEGG pathway analysis were calculated with the clusterProfiler package using the TCGA database. As expected, ubiquitin‐protein‐related activity and negative regulation of viral‐related processes were significantly regulated by the TRIM alterations in HCC (Figure 7C,D; Table S4). The GeneMANIA online tool was used to construct the network for the selected TRIMs with a network‐based gene‐ranking algorithm. The results showed that the four TRIM members were closely associated with genes involving tumor necrosis factor receptor super‐family binding, ubiquitin‐like protein ligase binding, cytokine receptor binding, and I‐kappaB kinase/NF‐kappB signaling (Figure 7E).

### 3.7 Associations between TRIM family genes, immune infiltration, and immune regulators

**FIGURE 8:**
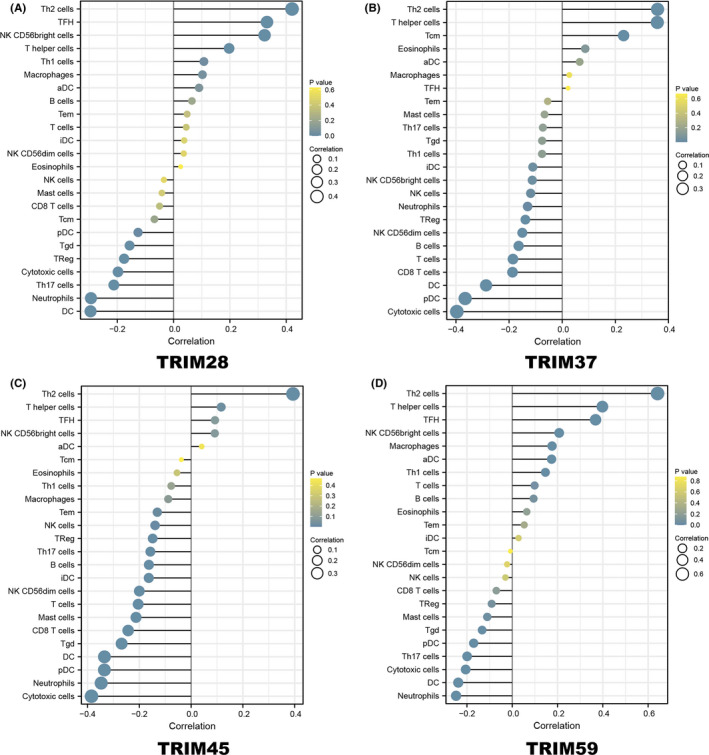
Association between the relative abundances of 24 immune cells and the expression level of (A) TRIM28, (B) TRIM37, (C) TRIM45 and (D) TRIM59 in HCC samples. The size of dots shows the absolute value of Spearman R

Accumulating evidence indicates that the immune microenvironment is key to modulating tumor progression and immunotherapy. Immunotherapy has revolutionized cancer therapy. Hence, we conducted further study of the relationships between TRIMs and immune cell infiltration level. In Figure 8A, the expression of TRIM28 was positively correlated with macrophages (Cor = 0.103, *p* = 0.048) and T helper cells, including Th1 (Cor = 0.108, *p* = 0.037) and Th2 (Cor = 0.421, *p* < 0.001) cells, and was negatively correlated with various immune cells including cytotoxic cells (Cor = −0.198, *p* < 0.001), dendritic cells (DC) (Cor = −0.295, *p* < 0.001), plasmacytoid DC (pDC) (Cor = −0.126, *p* = 0.015), neutrophils (Cor = −0.293, *p* < 0.001), and Treg (Cor = −0.176, *p* < 0.001). TRIM37 expression was positively associated with the infiltration of T helper cells (Cor = 0.358, *p* < 0.001), T central memory (Tcm) (Cor = 0.231, *p* < 0.001), and Th2 cells (Cor = 0.359, *p* < 0.001) and was negatively associated with various immune cells including B cells (Cor = −0.164, *p* = 0.001), CD8 T cells (Cor = −0.187, *p* < 0.001), T cells (Cor = −0.185, *p* < 0.001), cytotoxic cells (Cor = −0.397, *p* < 0.001), DC (Cor = −0.287, *p* < 0.001), immature DC (iDC) (Cor = −0.111, *p* < 0.001), pDC (Cor = −0.365, *p* < 0.001), neutrophils(Cor = −0.130, *p* = 0.012), and human natural killer (NK) cells such as NK CD56bright cells (Cor = −0.113, *p* = 0.029) and NK CD56dim cells (Cor = −0.150, *p =* 0.004) (Figure 8B). Similar results were found in TRIM45 and TRIM59 genes. We found positive correlations between TRIM45 and TRIM59 and the infiltration of Th2 cells, T follicular helpers, and NK CD56bright cells and significantly negative associations with neutrophils, Treg, Mast cells, Th17 cells, cytotoxic cells, DC, and pDC (Figure 8C,D).

### 3.8 GSEA identifies TRIMs‐related signaling pathways in ubiquitination and deubiquitination

**FIGURE 9:**
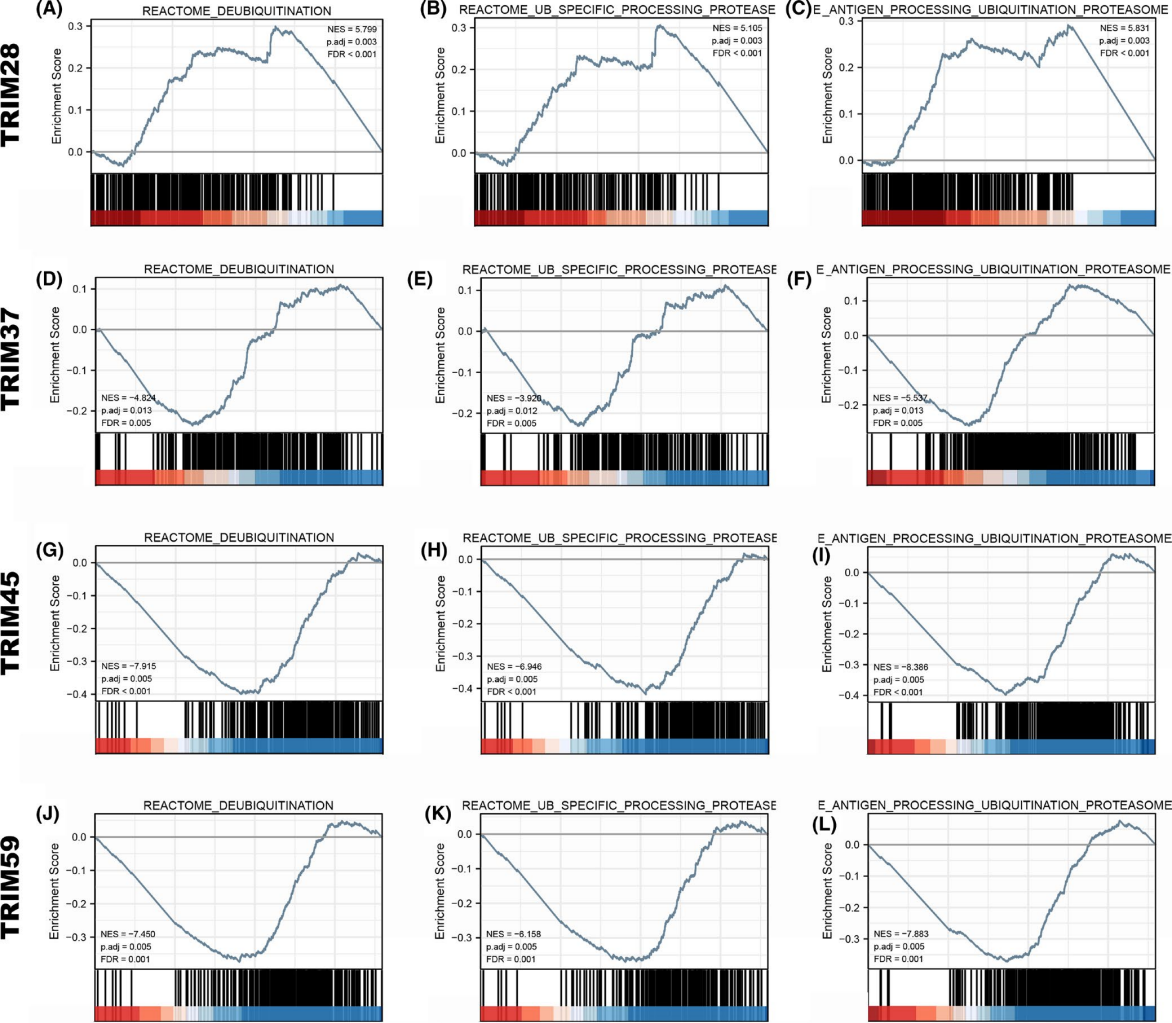
Enrichment plots from the gene set enrichment analysis (GSEA). Pathways and biological processes related to ubiquitination and deubiquitination were differentially enriched in (A–C) TRIM28, (D–F) TRIM37, (G–I) TRIM45 and (J–L) TRIM59 in HCC

The results showed that the HCC groups with high expression of TRIM28, TRIM37, TRIM45, and TRIM59 were significantly enriched in Reactome_UB_specific_processing_proteases (NES = 5.105, *p*. adjust = 0.003, FDR < 0.001; NES = −3.928, *p*. adjust = 0.012, FDR = 0.005; NES = −6.946, *p*. adjust = 0.005, FDR < 0.001; NES = −6.158, *p*. adjust = 0.005, FDR = 0.001, respectively), Reactome_antigen_processing_ubiquitination_proteasome_degradation (NES = 5.831, *p*. adjust = 0.003, FDR < 0.001; NES = −5.537, *p*. adjust = 0.013, FDR = 0.005; NES = −8.386, *p*. adjust = 0.005, FDR < 0.001; NES = −7.883, *p*. adjust = 0.005, FDR = 0.001, respectively), and Reactome_deubiquitination (NES = 5.799, *p*. adjust = 0.003, FDR < 0.001; NES = −4.824, *p*. adjust = 0.013, FDR = 0.005; NES = −7.915, *p*. adjust = 0.005, FDR < 0.001; NES = −7.450, *p*. adjust = 0.005, and FDR = 0.001, respectively) (Figure 9; Tables S5–S8).

## 4 DISCUSSION

To date, human HCC is characterized as the most common visceral neoplasms with a high mortality.^24^ Elucidating the molecular mechanism relevant to HCC carcinogenesis may help establish effective therapeutic targets or promising molecular biomarkers. However, the role of TRIMs in HCC warrants further investigation. The present study is the first to comprehensively explore the expression levels and potential prognostic values of members of the TRIM family in HCC. Through step‐by‐step exploration, we found that four genes were differentially overexpressed in HCC and were also statistically associated with various survival indicators. The data suggest that TRIM28, TRIM37, TRIM45, and TRIM59 were playing significant roles in HCC and may serve as promising prognostic biomarkers and therapeutic targets.

Among the TRIMs, several have been reported to have an antitumor effect in HCC (Table 3). Previous studies have elucidated that TRIM21, TRIM26, TRIM35, TRIM50, and TRIM56 acted as tumor suppressors by inhibiting tumor cell proliferation, and TRIM3, TRIM16, TRIM21, TRIM25, and TRIM26 negatively regulated migration and invasion in HCC cells. The results were reversed when the genes were knocked down or knocked out.^25^, ^26^, ^27^, ^28^, ^29^, ^30^, ^31^ Low expression levels of TRIM21, TRIM26, and TRIM35 had significantly shorter OS, and both TRIM3 and TRIM55 were independent factors positively affecting the prognosis of HCC patients.^26^, ^28^, ^32^, ^33^, ^34^ Different molecular mechanisms were proposed to participate in tumor cell regulation, including metabolic reprogramming, cell cycle arrest, phosphorylation, and ubiquitination.

**TABLE 3:** Studies reporting that TRIMs contribute to inhibit HCC tumor growth

Year	Author	TRIM member	Tumor model	Findings	Proposed mechanism
2014	Jie Chao et al^24^	TRIM3	43 paired cancerous and corresponding noncancerous tissues	Independent prognosis factor; increased 5‐year survival rate	—
2014	Zhiao Chen et al^25^	TRIM35	HCC tissues and adjacent nontumor liver tissues from 688 patients	Negative TRIM35 had shorter OS and TTR; downregulated in HCC cells and samples	Decreased Warburg effect; suppress tumorigenicity through the blockade of PKM2 Y105 phosphorylation
2015	Yi Wang et al^26^	TRIM26	HCC cell lines (HepG2, SMMC7721, Huh7, Bel‐7402, PLC, LM3, 97 L, 97H); 70 human samples	Low TRIM21 had shorter OS and RFS; downregulated in HCC cells and samples; TRIM26 silencing enhanced proliferation, migration, and invasion of HCC	Regulate cell metabolism and result in the metabolic reprogramming of cancer cell
2015	Qianshan Ding et al^27^	TRIM21	HepG2, PLC/PRF‐5, LM3, Huh7, SMMC7721, LM3, 97L, 97H; 70 human liver tissues	Low TRIM21 had shorter OS and RFS; downregulated in HCC cells and samples; silencing of TRIM21 promotes HCC colony forming, proliferation, migration, and antiapoptosis	IKK‐NF‐κB signaling
2015	Z Chen et al^28^	TRIM35	SMMC‐7721, Huh7, and SK‐Hep1; nude mice	Decreased in the Warburg effect and the cell proliferation of HCC cells	Inhibit tumorigenicity by blocking PKM2 Y105 phosphorylation
2016	LINGLIN LI et al^29^	TRIM16	HepG2, HCCLM3,SMMC‐7721, MHCC97H; 61 tumor and para‐cancerous tissues	Lower expression correlated with metastasis property; suppression of TRIM16 promotes migration and invasion; decreased EMT behavior	Decreased ZEB2 expression via a proteasome‐dependent pathway
2017	Xu‐Qiong Huang et al^30^	TRIM3	HepG2, Hep3B, and SK‐Hep1, Huh7 and Bel‐7402; female BALB/c nude mice	Decreased cell growth; decreased migration and invasion	Induced G0/G1 phase arrest
2017	HL Zang et al^31^	TRIM25	25 Chinese patients; HCC cell lines HuH6	Decreased migration and invasion	Decreased MTA1 protein expression
2018	Xiaoxiao Ma et al^32^	TRIM50	Three cohorts of 182 pairs of liver tissues; SMMC7721, BEL7402, HepG2, and HUH7; BALB/c athymic nude mice	Decreased contributed to progression; decreased proliferation, colony formation, and invasion; reversed anoikis resistance and induced apoptotic	Induced ubiquitous degradation of SNAIL by poly‐ubiquitination; reversed EMT
2019	Xinyu Li et al^33^	TRIM55	100 patients' HCC tissues and adjacent nontumor tissues; HCC cell lines HCC‐LM3 and Huh7	Independent prognosis factor; low TRIM55 had worse prognosis; decreases migration and invasion	Via EMT and MMP2
2020	Lihui Zhu et al^34^	TRIM7	Human HepG2 and Huh7; BALB/c athymic nude mice; 80 pairs of HCC tissues and corresponding noncancerous tissues	Decreased cell proliferation, invasion, and colony formation; decreased tumorigenesis; decreased HCC progression	Increased Lys48‐linked poly‐ubiquitination of Src via its RING domain; decreased Src‐mTORC1‐S6K1 axis
2021	Y. YANG et al^35^	TRIM56	Forty‐one paired HCC and para‐cancerous tissues; Bel‐7402, HepG2, MHCC88H, Bel‐7402, Huh7, Hep3B	Decreased viability, colony number, and EdU‐positive rate	Wnt signaling; inhibited by RBM24

Abbreviations: HCC, hepatocellular carcinom; OS, overall survival; RFS, relapse‐free survival; TRIM, TRIpartite motif; TTR, time‐to‐response.

However, more TRIMs were also stimulatory and cancerogenic factors in HCC (Table 4). The TRIM11, TRIM31, TRIM32, TRIM52, and TRIM59 expression levels were significantly upregulated in human HCC and acted as active regulators in promoting cell proliferation and in vivo tumorigenesis.^10^, ^11^, ^13^, ^35^, ^36^, ^37^, ^38^ As expected, downregulation of the above TRIMs markedly suppressed in vitro HCC cell proliferation and decreased the volume and weight of tumors in vivo. Except for the TRIM family members mentioned above, TRIM29, TRIM37, TRIM44, and TRIM65 facilitated HCC migration and invasion abilities in vitro.^39^, ^40^, ^41^, ^42^ Consistent with previous results TRIM11, TRIM29, TRIM32, TRIM44, and TRIM59 gene expression results, higher levels were significantly associated with poorer OS and other clinical outcomes.

**TABLE 4:** Studies reporting that TRIMs promote HCC carcinogenesis

Year	Author	TRIM member	Tumor model	Findings	Proposed mechanism
2015	Jianxin Jiang et al^40^	TRIM37	HCC cell line Hep3B, H7402, Huh7, SMMC7721, HepG2, SK‐Hep1, 97H, and LM3; tissue microarray of 90 HCC tissues	Worsened clinical outcomes; increased cell migration and tumor metastasis	Via Wnt/β‐catenin signaling
2016	Yanying Wang et al^44^	TRIM28/KAP1	Hepatoma cell lines HepG2, MHCC97L, and HCCLM3; 116 patients with HCC (74 men, 42 women)	Downregulation of KAP1 expression inhibits proliferation; decreased 5‐year survival rates; independent prognosis factor	Mediated by tumor suppressor gene p53
2016	Xiaopeng Cui et al^36^	TRIM32	HCC tissue microarrays; hepatoma carcinoma cell lines Huh7, HepG2, Hep3B	Worsened OS; downregulation of TRIM32 decreased cell proliferation rates and increased apoptosis after oxaliplatin exposure	Via G1‐S‐phase transition; cleaved caspase3
2016	Xinghua Zhu et al^42^	TRIM44	Tissue microarray of 106 matched pairs of primary HCC and adjacent noncancerous tissues; the human HCC cell lines (Huh7, HepG2, Hep3B)	Decreased OS; independent predictor of OS; increased cell proliferation; increased invasive and migratory capacity; enhanced doxorubicin resistance	Via accelerating the G1/S‐phase transition; through E‐ and N‐cadherin and vimentin; accelerating NF‐κB activation
2016	Yue Chen et al^56^	TRIM11	117 tumors and matched adjacent nontumor liver (NTL)	Decreased OS and DFS	
2017	Jinjin Liu et al^35^	TRIM11	10 HCC tissues; SMMC7721, MHCC97H, HepG2, and HCCLM3; BALB/c nude mice	Shortened HCC patient survival; increased proliferation, colony formation, migration, and invasion; increased tumor size; promotes EMT	Through E‐cadherin and vimentin; negatively regulating p53
2017	Yi Zhang et al^37^	TRIM11	20 pairs of HCC tissues and matched noncancerous tissues; MHCC97L, Huh1, and Hep3B; male BALB/c nude mice	Tumorigenesis; downregulation of TRIM11 inhibits the EMT	PI3K/Akt signaling pathway
2017	P Guo et al^55^	TRIM31	Paired HCC tissue and corresponding noncancerous liver tissues from 194 patients; HCC SMMC7721, HepG2， BEL7402, and Huh7	Increased proliferation, invasion, and colony formation; in vivo tumorigenicity	Increased mTORC1 pathway by negatively regulating TSC1–TSC2 complex through K48‐linked poly‐ubiquitous degradation
2017	Yi Zhang et al^37^	TRIM52	Peripheral blood samples; liver tissue samples; HepG2 and HepG2.2.15	TRIM52 silencing repressed the proliferation	Stimulate NF‐kB p65 expression
2017	Yu‐Feng Yang et al^41^	TRIM65	A cohort of 516 HCC cases; HCC cells (QGY‐7703 and Bel‐7404); male BALB/c nude mice	Independent prognosis factor; increased cell growth and migration	Increased β‐catenin signaling via ubiquitylation of Axin1
2018	Pengbo Guo et al^55^	TRIM31	SMMC7721, BEL7402, HepG2, and Huh7 cell lines	Increased anoikis resistance	Increased AMPK pathway by regulating p53 via ubiquitous modification
2018	Yi Zhang et al^11^	TRIM52	Tissue microarray of 87 HCC tissues; HCC cell line MHCC‐97H and MHCC‐97L	Increased cell proliferation; increased cell cycle progress, migration, and invasion	Decreased PPM1A by ubiquitination, increased MMP2, and induced Smad2/3 phosphorylation
2019	Huimin Du et al^39^	TRIM29	90 HCC tissues and paired normal adjacent tissues; HCC cell lines (MHCC‐97H, HepG2, SMMC‐7721, and Huh7) male BALB/c‐nu mice	Worsened OS; increased proliferation, migration, and invasion	Target gene of miR‐424‐5p; increased AFP, Bcl‐2, and Ki67 and decreased Bax
2019	Xia Hu et al^43^	TRIM7	84 patients' HCC and matched adjacent noncancerous tissue samples; HCC cell lines HepG2, MHCC‐97H and MHC‐97L	Worsened OS; knockdown‐inhibited cell growth	Via G1/S checkpoint; increased p38 phosphorylation via DUSP6
2019	Wanhu Fan et al^58^	TRIM66	Human HCC cell lines including Hep‐3B and SNU‐449; BALB/c athymic nude mice	Increased cell proliferation and invasion	Wnt/β‐catenin signaling
2020	Yanfeng Liu et al^12^	TRIM25	HCT116, U2OS, Huh7, MCF7, and MDA‐MB‐231 cells; athymic BALB/c nude mice	Required for ER homeostasis and negatively control UPR signaling pathway; increased tumor progression; worsened OS and DFS	Decreased Keap1 via ubiquitination; increased Nrf2 signaling pathway
2020	Peng Yuan et al^59^	TRIM25	25 pairs of liver samples of HCC and matched adjacent normal tissues; five HCC cell lines (SK‐HEP‐1, Hep3B, SMMC‐7721, HepG2, and Huh7); PDX model	Increased Epirubicin resistance	Via AKT signaling; modulating PTEN protein via ubiquitination
2020	Hanning Ying et al^38^	TRIM59	103 HCC tissues and paired adjacent nontumor tissue; HCC cell lines Huh7, HepG2, HCCLM3, Hep3B, and SK‐hep1; BALB/c nude mice	Worsened OS and higher recurrence probability; increased cell proliferation and migration	Decreased G1 phase; increased PPM1B ubiquitination through the RING domain
2021	Guosheng Tan et al^45^	TRIM37	53 HCC patients	Shorter survival time and an earlier relapse time; Sorafenib resistance	PI3K/Akt signaling

Abbreviations: DFS, disease‐free survival; HCC, hepatocellular carcinom; OS, overall survival; TRIM, TRIpartite motif.

Similar observations were reported in TRIM7 and TRIM25.^12^, ^43^ The expression levels of TRIM28, TRIM44, and TRIM65 were independent factors used to predict the survival rate of HCC patients.^41^, ^42^, ^44^ Moreover, further experiments were performed to assess the relationships between chemotherapy resistances and TRIM family member expression. The upregulation and prognostic potential outcomes in TRIM28, TRIM37, and TRIM59 were also observed in our study. Since TRIM32 was found to accelerate oxaliplatin resistance and TRIM44 to enhance the doxorubicin resistance of HCC cells in 2016, accumulating evidence has supported the causative drug‐resistance role in TRIM members and suggested targeting TRIM family proteins to augment the sensitivity of HCC toward chemotherapy.^12^, ^36^, ^42^, ^45^ Although they may have similar biologic characteristics, the underlying biologic mechanisms differ from one TRIM family member to another and remain poorly understood. The contribution of regulatory in cell signal transduction, ubiquitylation, phosphorylation, and other regulatory mechanisms need further verification.

Although immunotherapy has been a breakthrough in HCC treatment, immune checkpoint inhibitors benefit only a small percentage of patients.^46^ The role of the immune system in cancer growth must be clarified and reliable clinical markers for predicting immunotherapy responses explored. Tumor‐infiltrating lymphocytes (TILs) play key roles in tumorigenesis and neoplasm.^47^ TILs establish a complex intracellular network, maintaining and improving the immunosuppressive microenvironment, and promoting tumor immune escape, eventually leading to tumor progression.^48^ In the present manuscript, all four members selected from the TRIMs family were closely related to TILs. There were more TAMNK cells and fewer Th17 cells in the TRIM28‐, TRIM37‐, TRIM45‐, and TRIM59‐high HCC groups compared with the low expression groups, indicating that the downregulation of adaptive immunity contributed to an increasing in innate immunity. Previous studies have shown that TRIM28 is a negative immune regulator in response to various immune stimuli, and inhibition of TRIM28 leads to the synthesis of immunostimulatory cytokines and activation of anticancer responses.^49^, ^50^ Consistently, TRIM28 knockout in melanomas was sufficient to activate immune infiltration.^51^ The negative correlation between TRIM28 and immune infiltration has been reported in lung adenocarcinoma based on the Estimation of STromal and Immune cells in MAlignant Tumor tissues using Expression data (ESTIMATE) algorithm in regulating the levels of diverse chemokines and molecular signaling pathways.^52^ Based on the involvement of TRIM37 in T lymphocyte derangement in MUL syndrome, targeting TRIM37 will reveal the importance of T cell function, particularly in CD4^+^ lymphocytes.^53^ Collectively, the data provide a rational to develop TRIMs‐targeted small molecules to enhance therapeutic effects.

To further investigate the biologic functions of TRIMs selected as pivotal genes in HCC, we performed KEGG, GO, and GESA analyses. The results showed that 88.1% of the significantly enriched pathways were common among the four TRIM members, notably, ubiquitination and deubiquitination. Interleukin signaling, growth factor receptor signaling, cell cycle regulation pathway, MAPK family signaling, Wnt signaling, and nuclear receptor pathway were enriched in the TRIM28‐, TRIM37‐, TRIM45‐, and TRIM59‐high phenotype. The RING‐finger domain of the TRIM family members harbors E3 ubiquitin ligase activity and confers the ubiquitous activations to their target proteins.^9^ As HCC suppressor genes, TRIM16 regulates ZEB2 proteins through ubiquitn‐proteasome pathway to inhibit EMT behavior and the number of distant metastasis tumors, which could be completely abrogated by proteasome inhibition.^27^ Through immunoprecipitation and a ubiquitination assay, it was verified that TRIM50 could induce poly‐ubiquitination of the SNAIL protein in both the nuclei and cytoplasmic compartments. The RING domain deleted mutant significantly rescued the negative degradation of SNAIL.^31^ Similar mechanisms were also detected in TRIM7 reducing Src protein.^54^

As for the TRIM oncogenes in HCC, TRIM65 triggered β‐catenin signaling via ubiquitylation of the Axin1 protein. TRIM52 significantly modulated the ubiquitination of PPM1A in HCC cells to exert carcinogenic functions.^11^, ^41^ Using integrated investigation systems, including cellular models, animal models, and clinical liver cancer specimens, TRIM31 promoted HCC progression by inducing K48‐linked poly‐ubiquitous degradation of the tuberous sclerosis complex (TSC), the 1‐TSC2 complex, and the p53‐AMPK axis to mediate anoikis resistance.^10^, ^55^ Furthermore, a ubiquitous analysis showed that TRIM25 poly‐ubiquitinated the metastasis‐associated 1 protein (MTA1) and participated in Keap1 degradation by the ubiquitin‐proteasome pathway.^12^, ^30^ The latter could be reversed by a series of truncated mutants of TRIM25, including three truncated mutants and Glu9 and Glu10 mutations. However, the mechanisms underlying the E3 ubiquitin ligase activities require further investigation.

We comprehensively analyzed the expression and prognostic value of TRIM family proteins in liver cancer and provided a thorough understanding of the heterogeneity of HCC's molecular biologic properties. Our results indicated that the increased expression of TRIM28, TRIM37, TRIM45, and TRIM59 in liver cancer tissues likely plays a pivot role in HCC. High expression of these four could also serve as biomarkers to identify high‐risk subgroups and potential treatment targets to enhance the prognosis of HCC patients.

## CONFLICT OF INTEREST

The authors report no conflicts of interest.

## AUTHOR CONTRIBUTIONS

Methodology: Lingyun Wu and Kan Jiang; Data acquisition: Hao Yu and Lingling Yang; Writing‐original draft: Lingyun Wu and Xin Yin; Revised the manuscript: Lingyun Wu and Jie Yin; Writing—review and editing: all authors; Supervision: Senxiang Yan and Chiyuan Ma.

## ETHICS STATEMENT

Not applicable.

## CONSENT FOR PUBLICATION

Written informed consent for publication was obtained from all participants.

## Supporting information


Figure S1

Figure S2

Table S1

Table S2

Table S3

Table S4

Table S5
Table S6
Table S7
Table S8

## Data Availability

The datasets analyzed during our study are available from the corresponding author on reasonable request.

## References

[1] Sung H , Ferlay J , Siegel RL , et al. Global cancer statistics 2020: GLOBOCAN estimates of incidence and mortality worldwide for 36 cancers in 185 countries. CA Cancer J Clin. 2021;71:209‐249. 33538338 10.3322/caac.21660

[2] Villanueva A . Hepatocellular carcinoma. N Engl J Med. 2019;380:1450‐1462. 30970190 10.1056/NEJMra1713263

[3] Hester CA , Yopp AC . Surgical therapies in hepatocellular carcinoma. In: Hoshida Y , ed. Hepatocellular Carcinoma: Translational Precision Medicine Approachesed. Humana Press; 2019:145‐167. 32078277

[4] Davila JA , Duan Z , McGlynn KA , El‐Serag HB . Utilization and outcomes of palliative therapy for hepatocellular carcinoma: a population‐based study in the United States. J Clin Gastroenterol. 2012;46:71‐77. 22157221 10.1097/MCG.0b013e318224d669 PMC3832893

[5] Reymond A , Meroni G , Fantozzi A , et al. The tripartite motif family identifies cell compartments. EMBO J. 2001;20:2140‐2151. 11331580 10.1093/emboj/20.9.2140 PMC125245

[6] Avela K , Lipsanen‐Nyman M , Idanheimo N , et al. Gene encoding a new RING‐B‐box‐coiled‐coil protein is mutated in mulibrey nanism. Nat Genet. 2000;25:298‐301. 10888877 10.1038/77053

[7] Hatakeyama S . TRIM family proteins: roles in autophagy, immunity, and carcinogenesis. Trends Biochem Sci. 2017;42:297‐311. 28118948 10.1016/j.tibs.2017.01.002

[8] Huibregtse JM , Scheffner M , Beaudenon S , Howley PM . A family of proteins structurally and functionally related to the E6‐AP ubiquitin‐protein ligase. Proc Natl Acad Sci U S A. 1995;92:5249. 7761480 10.1073/pnas.92.11.5249-b PMC55685

[9] Pauletto E , Eickhoff N , Padrao NA , Blattner C , Zwart W . TRIMming down hormone‐driven cancers: the biological impact of TRIM proteins on tumor development, progression and prognostication. Cell. 2021;10:1517. 10.3390/cells10061517 PMC8234875 34208621

[10] Guo P , Ma X , Zhao W , et al. TRIM31 is upregulated in hepatocellular carcinoma and promotes disease progression by inducing ubiquitination of TSC1‐TSC2 complex. Oncogene. 2018;37:478‐488. 28967907 10.1038/onc.2017.349

[11] Zhang Y , Tao R , Wu SS , et al. TRIM52 up‐regulation in hepatocellular carcinoma cells promotes proliferation, migration and invasion through the ubiquitination of PPM1A. J Exp Clin Cancer Res. 2018;37:116. 29898761 10.1186/s13046-018-0780-9 PMC6001170

[12] Liu Y , Tao S , Liao L , et al. TRIM25 promotes the cell survival and growth of hepatocellular carcinoma through targeting Keap1‐Nrf2 pathway. Nat Commun. 2020;11:348. 31953436 10.1038/s41467-019-14190-2 PMC6969153

[13] Zhang Z , Xu C , Zhang X , et al. TRIM11 upregulation contributes to proliferation, invasion, and EMT of hepatocellular carcinoma cells. Oncol Res. 2017;25:691‐699. 28244856 10.3727/096504016X14774897404770 PMC7841231

[14] Rhodes DR , Kalyana‐Sundaram S , Mahavisno V , et al. Oncomine 3.0: genes, pathways, and networks in a collection of 18,000 cancer gene expression profiles. Neoplasia. 2007;9:166‐180. 17356713 10.1593/neo.07112 PMC1813932

[15] Tang Z , Li C , Kang B , Gao G , Li C , Zhang Z . GEPIA: a web server for cancer and normal gene expression profiling and interactive analyses. Nucleic Acids Res. 2017;45:W98‐W102. 28407145 10.1093/nar/gkx247 PMC5570223

[16] Gao J , Aksoy BA , Dogrusoz U , et al. Integrative analysis of complex cancer genomics and clinical profiles using the cBioPortal. Sci Signal. 2013;6:pl1. 23550210 10.1126/scisignal.2004088 PMC4160307

[17] Wu L , Zhu X , Yan D , Tang M , Ma C , Yan S . Identification of IFN‐induced transmembrane protein 1 with prognostic value in pancreatic cancer using network module‐based analysis. Front Oncol. 2021;11:626883. 33869009 10.3389/fonc.2021.626883 PMC8044951

[18] Li T , Fu J , Zeng Z , et al. TIMER2.0 for analysis of tumor‐infiltrating immune cells. Nucleic Acids Res. 2020;48:W509‐W514. 32442275 10.1093/nar/gkaa407 PMC7319575

[19] Wurmbach E , Chen YB , Khitrov G , et al. Genome‐wide molecular profiles of HCV‐induced dysplasia and hepatocellular carcinoma. Hepatology. 2007;45:938‐947. 17393520 10.1002/hep.21622

[20] Chen X , Cheung ST , So S , et al. Gene expression patterns in human liver cancers. Mol Biol Cell. 2002;13:1929‐1939. 12058060 10.1091/mbc.02-02-0023. PMC117615

[21] Mas VR , Maluf DG , Archer KJ , et al. Genes involved in viral carcinogenesis and tumor initiation in hepatitis C virus‐induced hepatocellular carcinoma. Mol Med. 2009;15:85‐94. 19098997 10.2119/molmed.2008.00110 PMC2605622

[22] Roessler S , Jia HL , Budhu A , et al. A unique metastasis gene signature enables prediction of tumor relapse in early‐stage hepatocellular carcinoma patients. Cancer Res. 2010;70:10202‐10212. 21159642 10.1158/0008-5472.CAN-10-2607 PMC3064515

[23] Guichard C , Amaddeo G , Imbeaud S , et al. Integrated analysis of somatic mutations and focal copy‐number changes identifies key genes and pathways in hepatocellular carcinoma. Nat Genet. 2012;44:694‐698. 22561517 10.1038/ng.2256 PMC3819251

[24] Xu Q , Wang Y , Huang W . Identification of immune‐related lncRNA signature for predicting immune checkpoint blockade and prognosis in hepatocellular carcinoma. Int Immunopharmacol. 2021;92:107333. 33486322 10.1016/j.intimp.2020.107333

[25] Chen Z , Wang Z , Guo W , et al. TRIM35 interacts with pyruvate kinase isoform M2 to suppress the Warburg effect and tumorigenicity in hepatocellular carcinoma. Oncogene. 2015;34:3946‐3956. 25263439 10.1038/onc.2014.325

[26] Ding Q , He D , He K , et al. Downregulation of TRIM21 contributes to hepatocellular carcinoma carcinogenesis and indicates poor prognosis of cancers. Tumour Biol. 2015;36:8761‐8772. 26055142 10.1007/s13277-015-3572-2

[27] Li L , Dong L , Qu X , Jin S , Lv X , Tan G . Tripartite motif 16 inhibits hepatocellular carcinoma cell migration and invasion. Int J Oncol. 2016;48:1639‐1649. 26892350 10.3892/ijo.2016.3398

[28] Wang Y , He D , Yang L , et al. TRIM26 functions as a novel tumor suppressor of hepatocellular carcinoma and its downregulation contributes to worse prognosis. Biochem Biophys Res Commun. 2015;463:458‐465. 26043685 10.1016/j.bbrc.2015.05.117

[29] Huang XQ , Zhang XF , Xia JH , et al. Tripartite motif‐containing 3 (TRIM3) inhibits tumor growth and metastasis of liver cancer. Chin J Cancer. 2017;36:77. 28950898 10.1186/s40880-017-0240-5 PMC5615435

[30] Zang HL , Ren SN , Cao H , Tian XF . The ubiquitin ligase TRIM25 inhibits hepatocellular carcinoma progression by targeting metastasis associated 1 protein. IUBMB Life. 2017;69:795‐801. 28861931 10.1002/iub.1661

[31] Ma X , Ma X , Qiu Y , et al. TRIM50 suppressed hepatocarcinoma progression through directly targeting SNAIL for ubiquitous degradation. Cell Death Dis. 2018;9:608. 29789583 10.1038/s41419-018-0644-4 PMC5964248

[32] Chen Z , Lu X , Wang Z , et al. Co‐expression of PKM2 and TRIM35 predicts survival and recurrence in hepatocellular carcinoma. Oncotarget. 2015;6:2538‐2548. 25576919 10.18632/oncotarget.2991 PMC4385869

[33] Chao J , Zhang XF , Pan QZ , et al. Decreased expression of TRIM3 is associated with poor prognosis in patients with primary hepatocellular carcinoma. Med Oncol. 2014;31:102. 24994609 10.1007/s12032-014-0102-9

[34] Li X , Huang L , Gao W . Overexpression of tripartite motif Conaining 55 (TRIM55) inhibits migration and invasion of hepatocellular carcinoma (HCC) cells via epithelial‐mesenchymal transition and matrix Metalloproteinase‐2 (MMP2). Med Sci Monit. 2019;25:771‐777. 30685767 10.12659/MSM.910984 PMC6360872

[35] Liu J , Rao J , Lou X , Zhai J , Ni Z , Wang X . Upregulated TRIM11 exerts its oncogenic effects in hepatocellular carcinoma through inhibition of P53. Cell Physiol Biochem. 2017;44:255‐266. 29190611 10.1159/000484678

[36] Cui X , Lin Z , Chen Y , et al. Upregulated TRIM32 correlates with enhanced cell proliferation and poor prognosis in hepatocellular carcinoma. Mol Cell Biochem. 2016;421:127‐137. 27573002 10.1007/s11010-016-2793-z

[37] Zhang Y , Wu S‐S , Chen X‐H , Tang Z‐H , Yu Y‐S , Zang G‐Q . Tripartite motif containing 52 (TRIM52) promotes cell proliferation in hepatitis B virus‐associated hepatocellular carcinoma. Med Sci Monit. 2017;23:5202‐5210. doi:10.12659/msm.907242 29089476 PMC5678430

[38] Ying H , Ji L , Xu Z , et al. TRIM59 promotes tumor growth in hepatocellular carcinoma and regulates the cell cycle by degradation of protein phosphatase 1B. Cancer Lett. 2020;473:13‐24. 31875525 10.1016/j.canlet.2019.12.030

[39] Du H , Xu Q , Xiao S , et al. MicroRNA‐424‐5p acts as a potential biomarker and inhibits proliferation and invasion in hepatocellular carcinoma by targeting TRIM29. Life Sci. 2019;224:1‐11. 30876939 10.1016/j.lfs.2019.03.028

[40] Jiang J , Yu C , Chen M , Tian S , Sun C . Over‐expression of TRIM37 promotes cell migration and metastasis in hepatocellular carcinoma by activating Wnt/beta‐catenin signaling. Biochem Biophys Res Commun. 2015;464:1120‐1127. 26208456 10.1016/j.bbrc.2015.07.089

[41] Yang YF , Zhang MF , Tian QH , Zhang CZ . TRIM65 triggers beta‐catenin signaling via ubiquitylation of Axin1 to promote hepatocellular carcinoma. J Cell Sci. 2017;130:3108‐3115. 28754688 10.1242/jcs.206623

[42] Zhu X , Wu Y , Miao X , et al. High expression of TRIM44 is associated with enhanced cell proliferation, migration, invasion, and resistance to doxorubicin in hepatocellular carcinoma. Tumour Biol. 2016;37:14615‐14628. 27619678 10.1007/s13277-016-5316-3

[43] Hu X , Tang Z , Ma S , Yu Y , Chen X , Zang G . Tripartite motif‐containing protein 7 regulates hepatocellular carcinoma cell proliferation via the DUSP6/p38 pathway. Biochem Biophys Res Commun. 2019;511:889‐895. 30850165 10.1016/j.bbrc.2019.02.001

[44] Wang Y , Jiang J , Li Q , Ma H , Xu Z , Gao Y . KAP1 is overexpressed in hepatocellular carcinoma and its clinical significance. Int J Clin Oncol. 2016;21:927‐933. 27095111 10.1007/s10147-016-0979-8

[45] Tan G , Xie B , Yu N , et al. TRIM37 overexpression is associated with chemoresistance in hepatocellular carcinoma via activating the AKT signaling pathway. Int J Clin Oncol. 2021;26:532‐542. 33387087 10.1007/s10147-020-01832-5

[46] Zheng Y , Li Y , Feng J , et al. Cellular based immunotherapy for primary liver cancer. J Exp Clin Cancer Res. 2021;40:250. 34372912 10.1186/s13046-021-02030-5 PMC8351445

[47] Demaria O , Vivier E . Immuno‐oncology beyond TILs: unleashing TILCs. Cancer Cell. 2020;37:428‐430. 32289267 10.1016/j.ccell.2020.03.021

[48] Yu YR , Imrichova H , Wang H , et al. Disturbed mitochondrial dynamics in CD8^+^ TILs reinforce T cell exhaustion. Nat Immunol. 2020;21:1540‐1551. 33020660 10.1038/s41590-020-0793-3

[49] Krischuns T , Gunl F , Henschel L , et al. Phosphorylation of TRIM28 enhances the expression of IFN‐beta and Proinflammatory cytokines during HPAIV infection of human lung epithelial cells. Front Immunol. 2018;9:2229. 30323812 10.3389/fimmu.2018.02229 PMC6172303

[50] Park HH , Kim HR , Park SY , et al. RIPK3 activation induces TRIM28 derepression in cancer cells and enhances the anti‐tumor microenvironment. Mol Cancer. 2021;20:107. 34419074 10.1186/s12943-021-01399-3 PMC8379748

[51] Lee AK , Pan D , Bao X , Hu M , Li F , Li CY . Endogenous retrovirus activation as a key mechanism of anti‐tumor immune response in radiotherapy. Radiat Res. 2020;193:305‐317. 32074012 10.1667/RADE-20-00013 PMC7359417

[52] Liu J , Han X , Chen L , et al. TRIM28 is a distinct prognostic biomarker that worsens the tumor immune microenvironment in lung adenocarcinoma. Aging. 2020;12:20308‐20331. 33091876 10.18632/aging.103804 PMC7655206

[53] Bruzzaniti S , Cirillo E , Prencipe R , et al. CD4^+^ T cell defects in a Mulibrey patient with specific TRIM37 mutations. Front Immunol. 2020;11:1742. 33042106 10.3389/fimmu.2020.01742 PMC7530177

[54] Zhu L , Qin C , Li T , et al. The E3 ubiquitin ligase TRIM7 suppressed hepatocellular carcinoma progression by directly targeting Src protein. Cell Death Differ. 2020;27:1819‐1831. 31802035 10.1038/s41418-019-0464-9 PMC7244582

[55] Guo P , Qiu Y , Ma X , et al. Tripartite motif 31 promotes resistance to anoikis of hepatocarcinoma cells through regulation of p53‐AMPK axis. Exp Cell Res. 2018;368:59‐66. 29665353 10.1016/j.yexcr.2018.04.013

[56] Chen Y , Li L , Qian X , Ge Y , Xu G . High expression of TRIM11 correlates with poor prognosis in patients with hepatocellular carcinoma. Clin Res Hepatol Gastroenterol. 2017;41(2):190‐196. doi:10.1016/j.clinre.2016.09.010 28065743

[57] Fan W , Du F , Liu X . TRIM66 confers tumorigenicity of hepatocellular carcinoma cells by regulating GSK‐3β‐dependent Wnt/β‐catenin signaling. Eur J Pharmacol. 2019;850:109–117. doi:10.1016/j.ejphar.2019.01.054 30710548

[58] Yuan P , Zheng A , Tang Q . Tripartite motif protein 25 is associated with epirubicin resistance in hepatocellular carcinoma cells via regulating PTEN/AKT pathway. Cell Biol Int. 2020;44(7):1503–1513. doi:10.1002/cbin.11346 32196840

