# Supplementary figures and images for: Comprehensive profiling of the TRIpartite motif family to identify pivot genes in hepatocellular carcinoma
Source: Cancer Med. 2022 Feb 9;11(7):1712–31. doi: 10.1002/cam4.4552 (PMC8986146; doi:10.1002/cam4.4552)

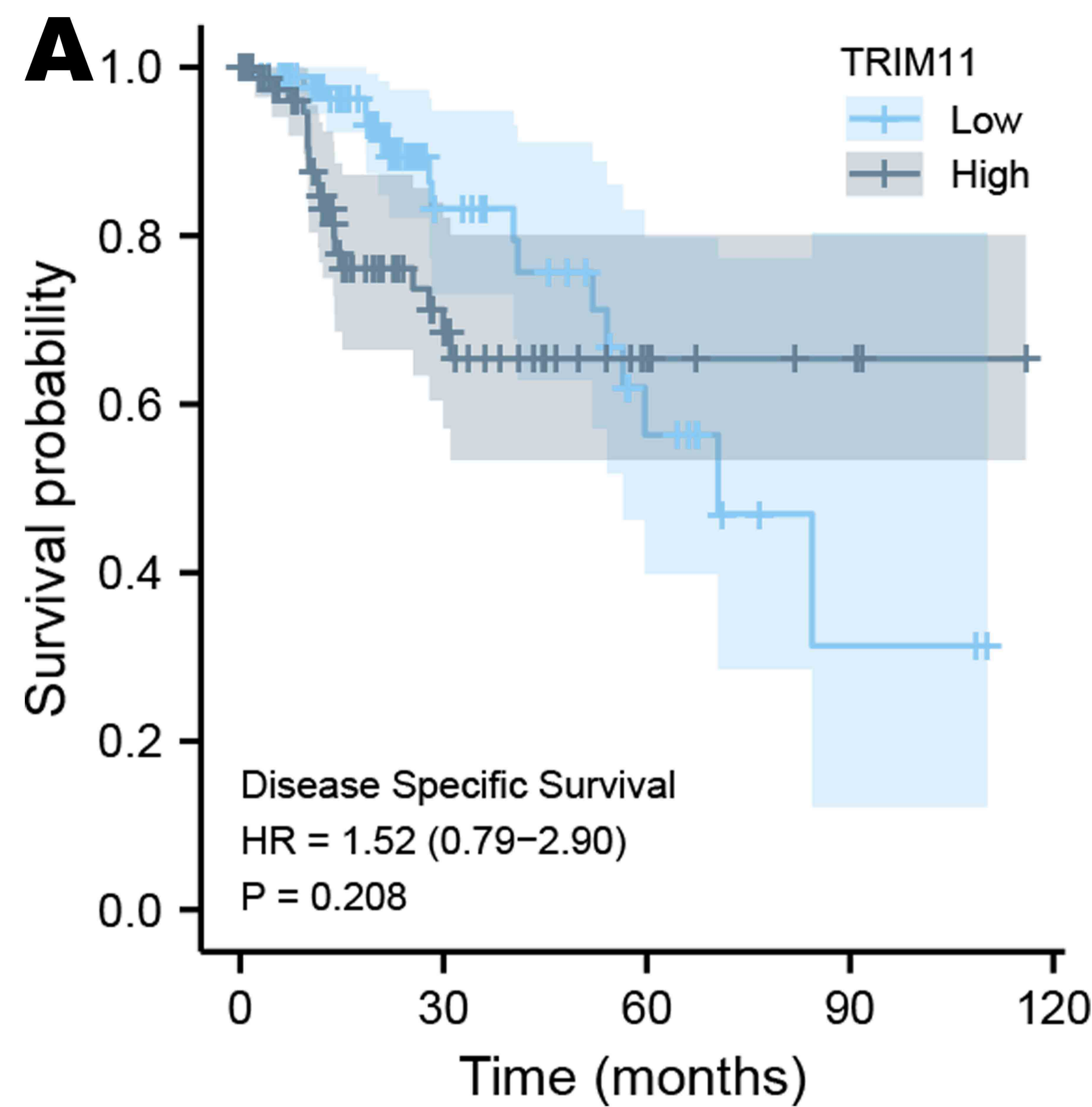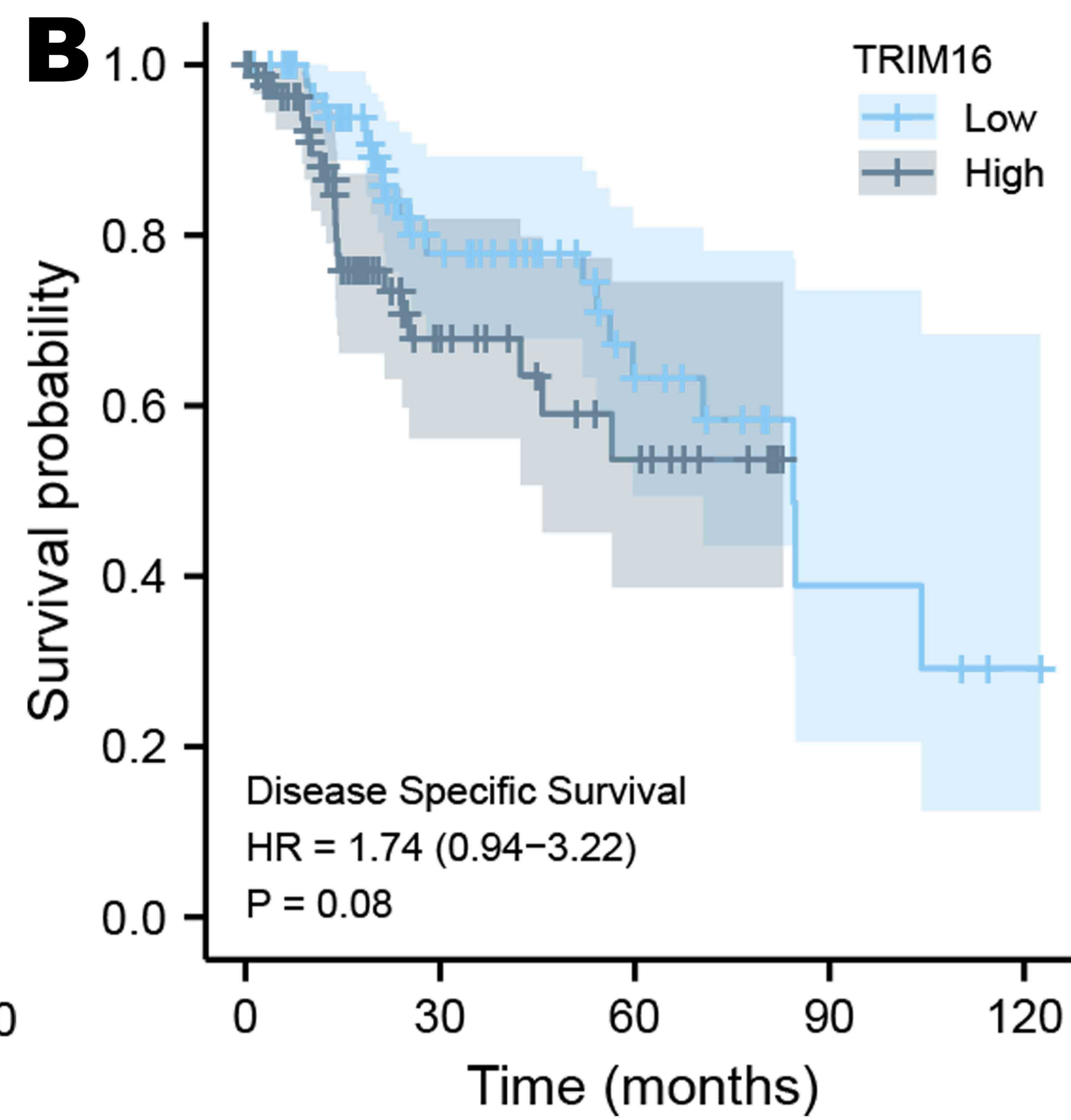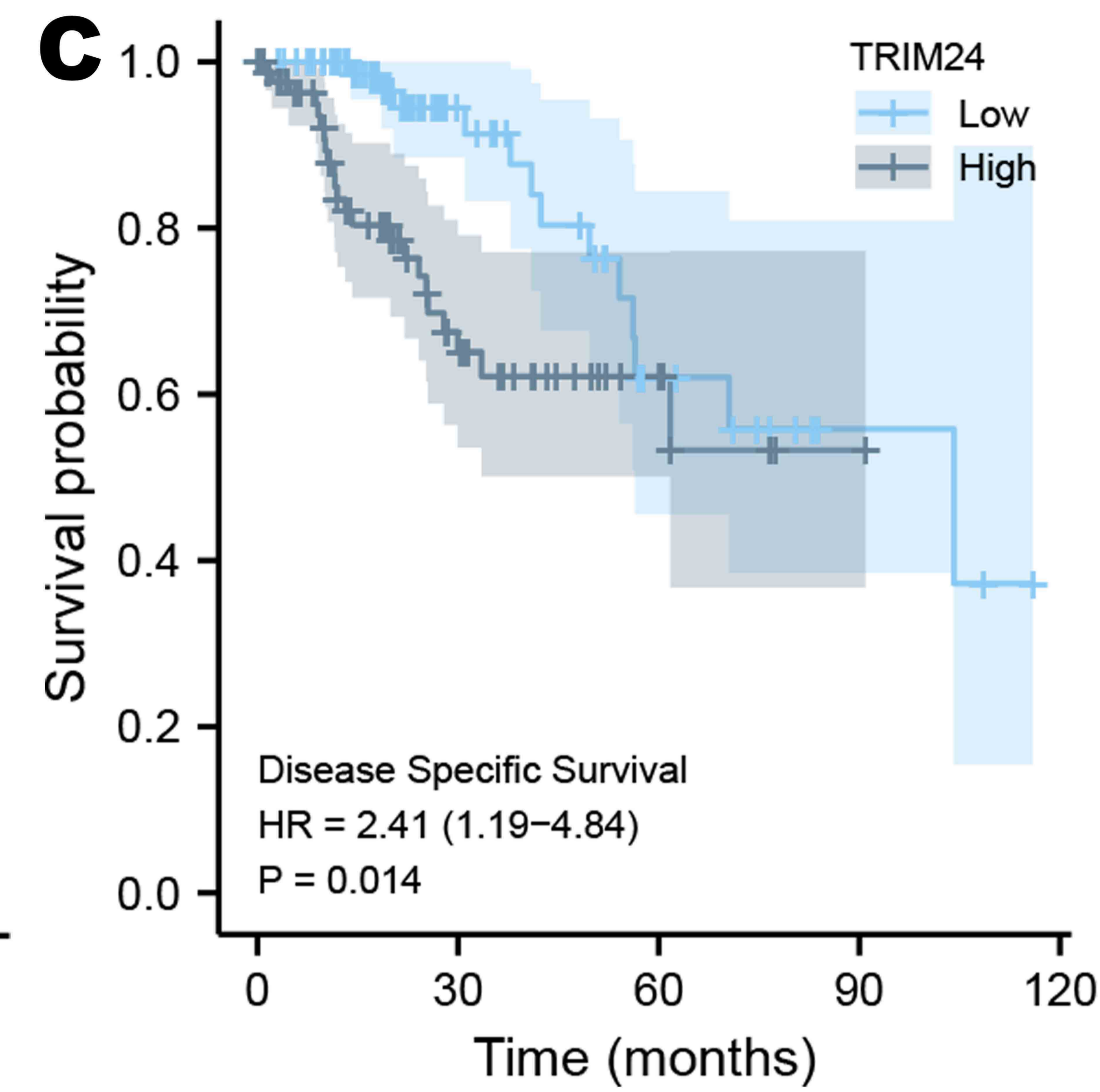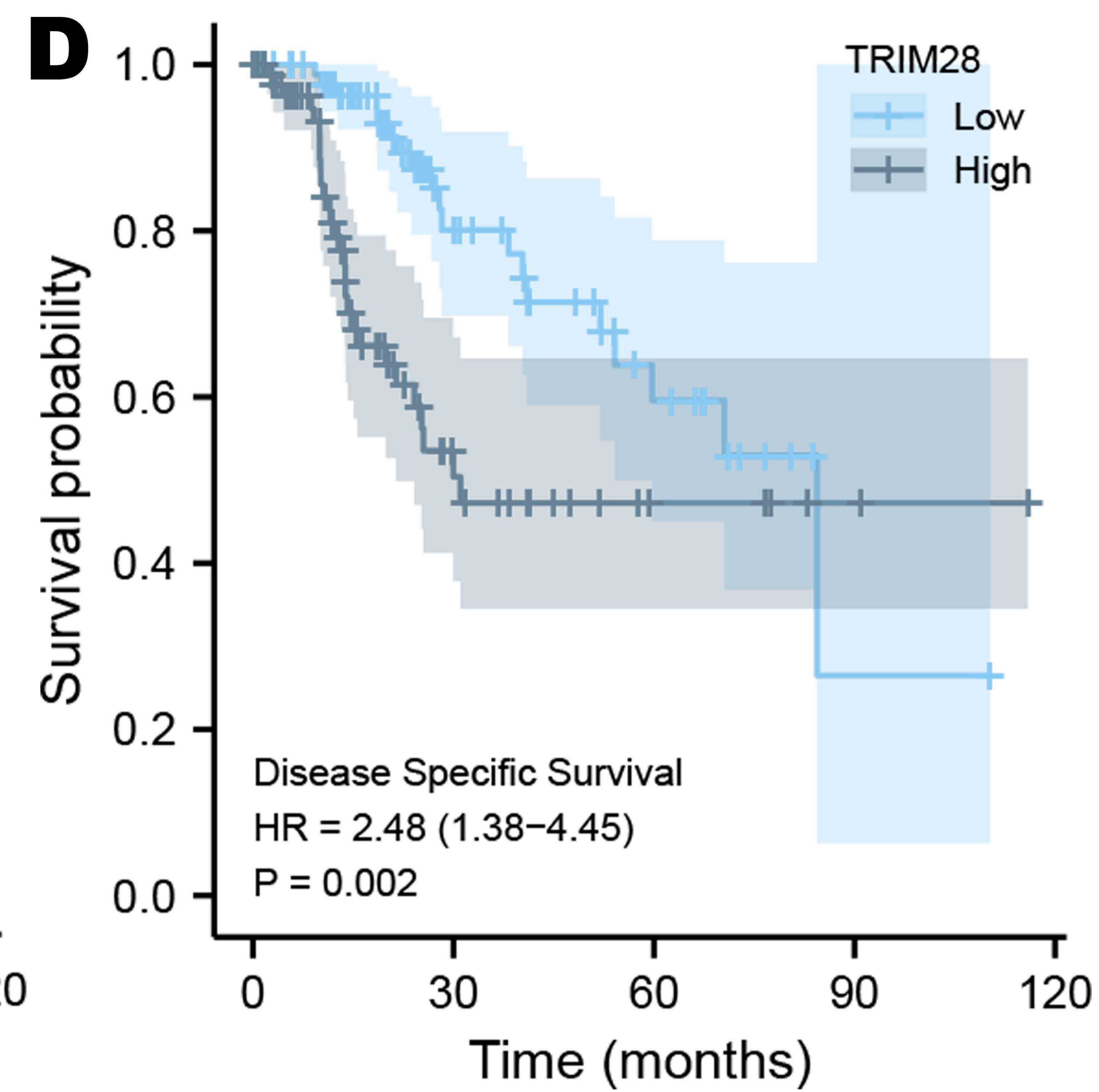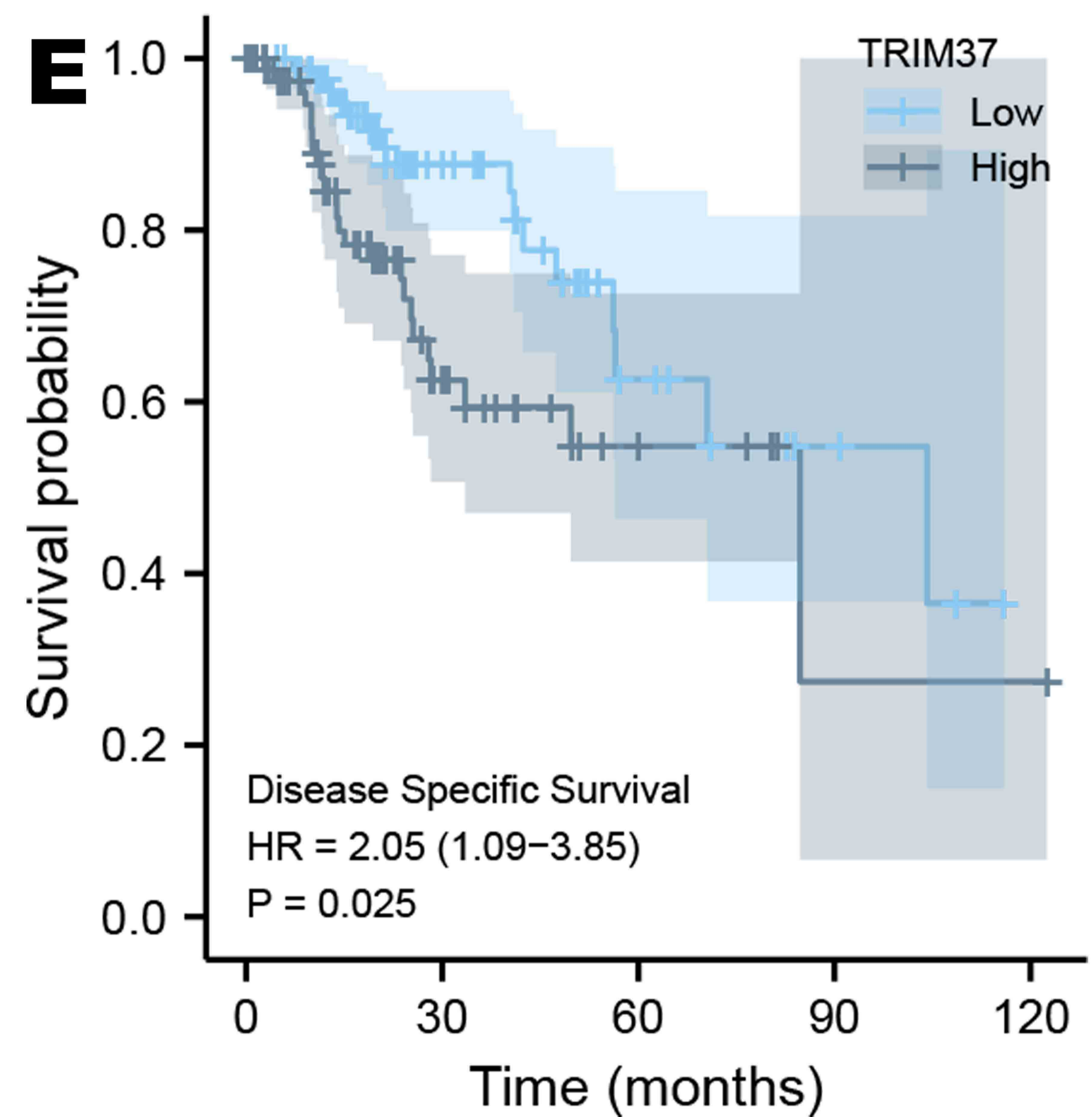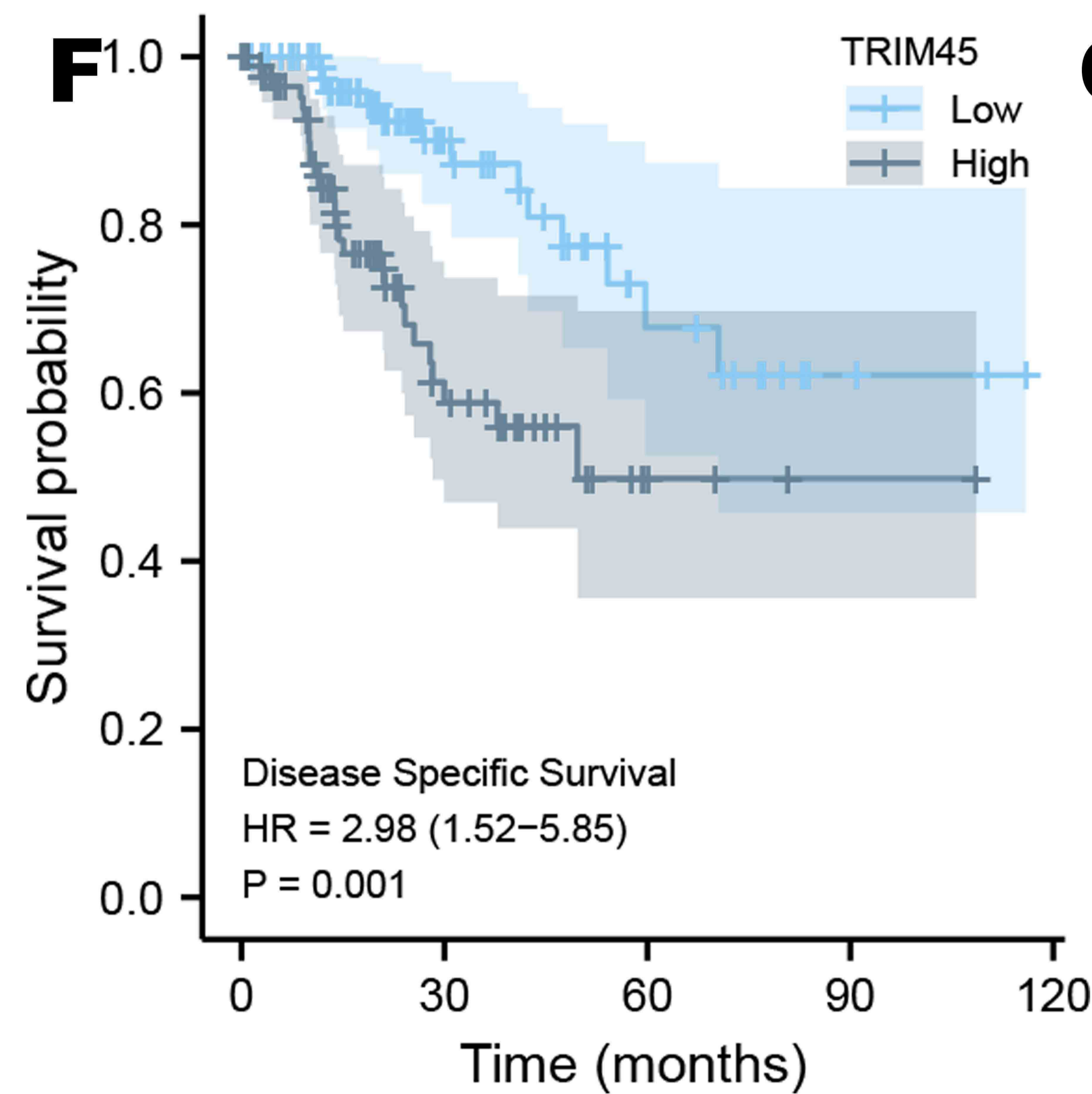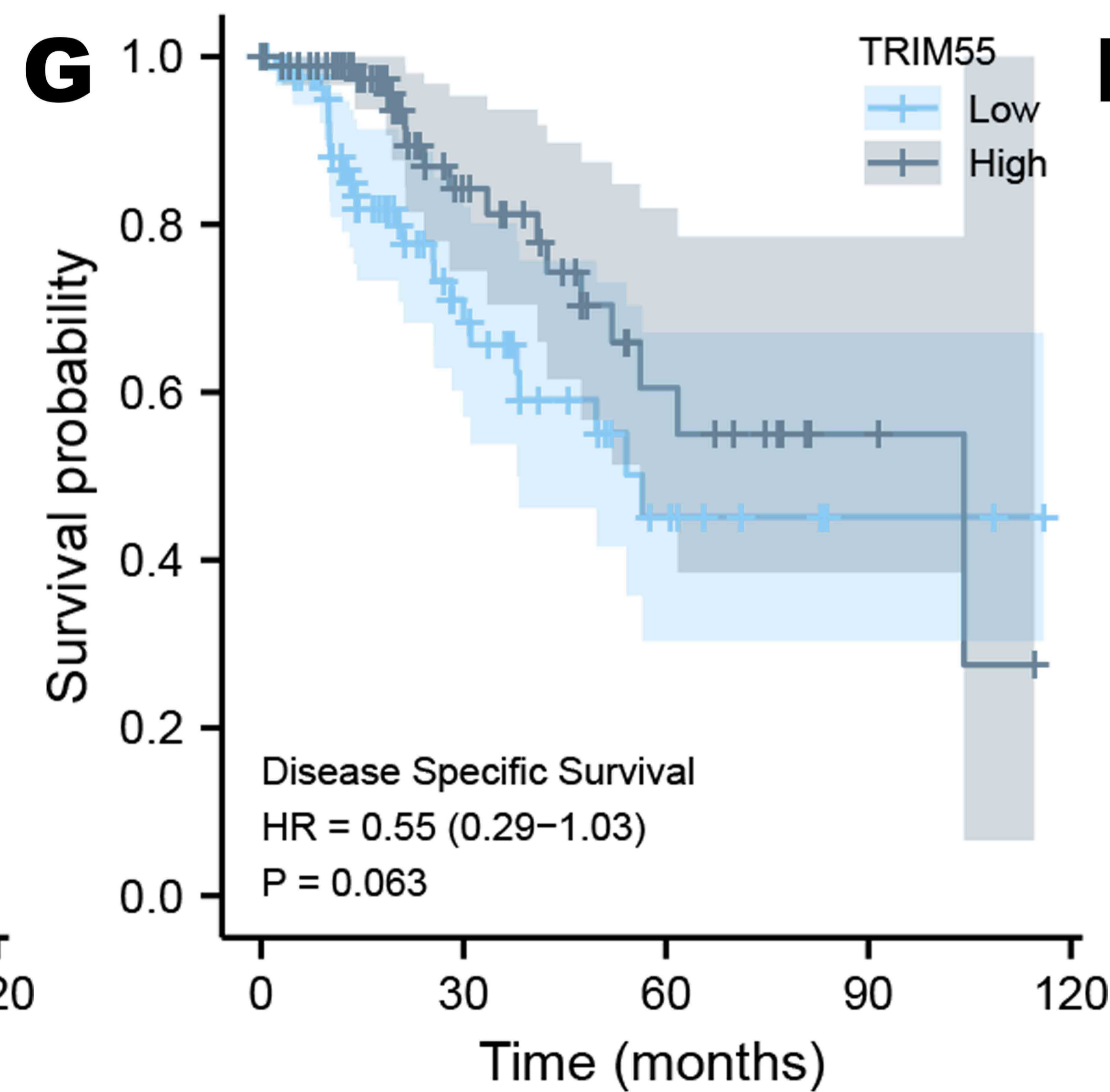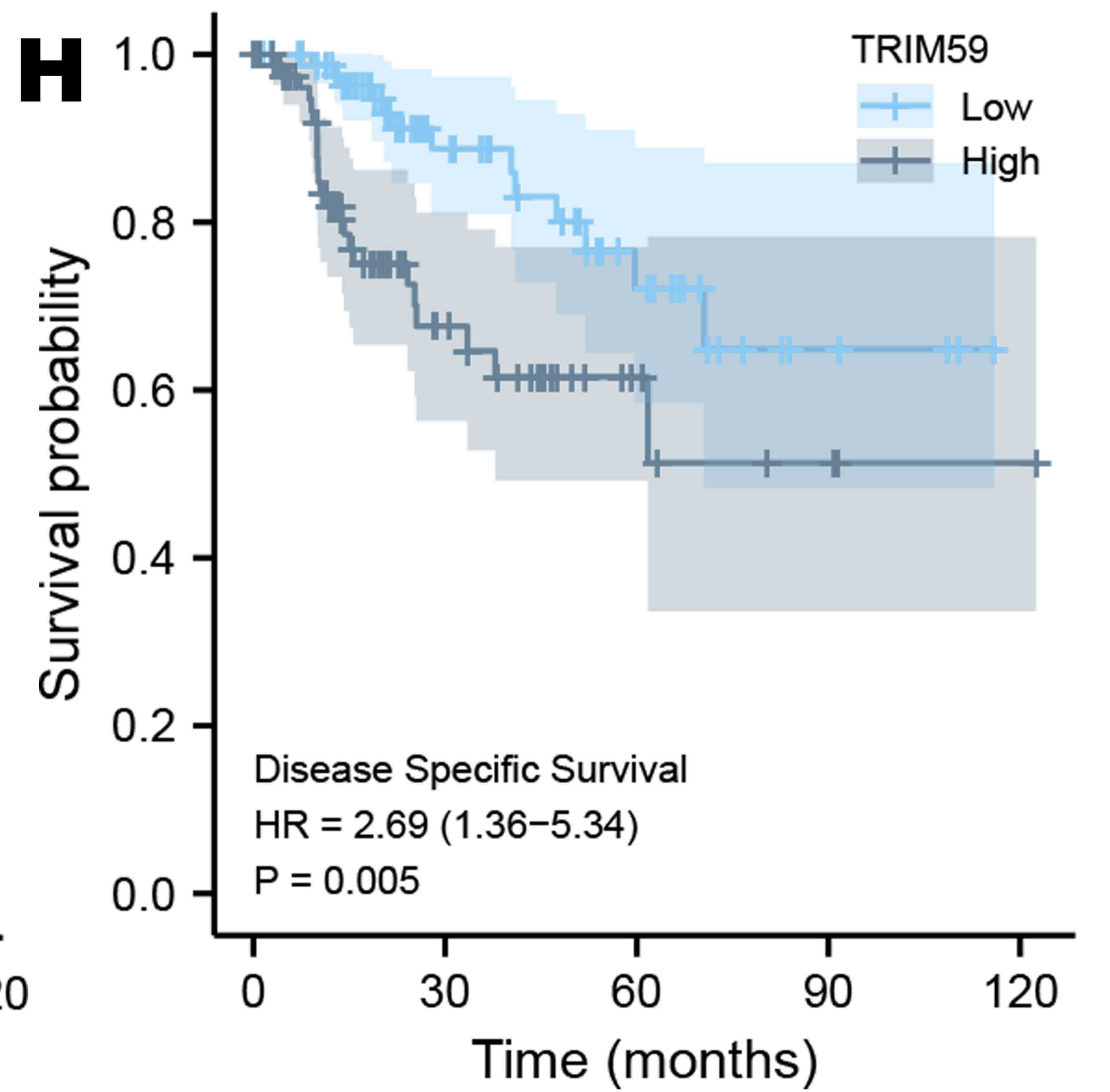

Supplement: Supplementary file 1 — Figure S1 [file CAM4-11-1712-s004.pdf]

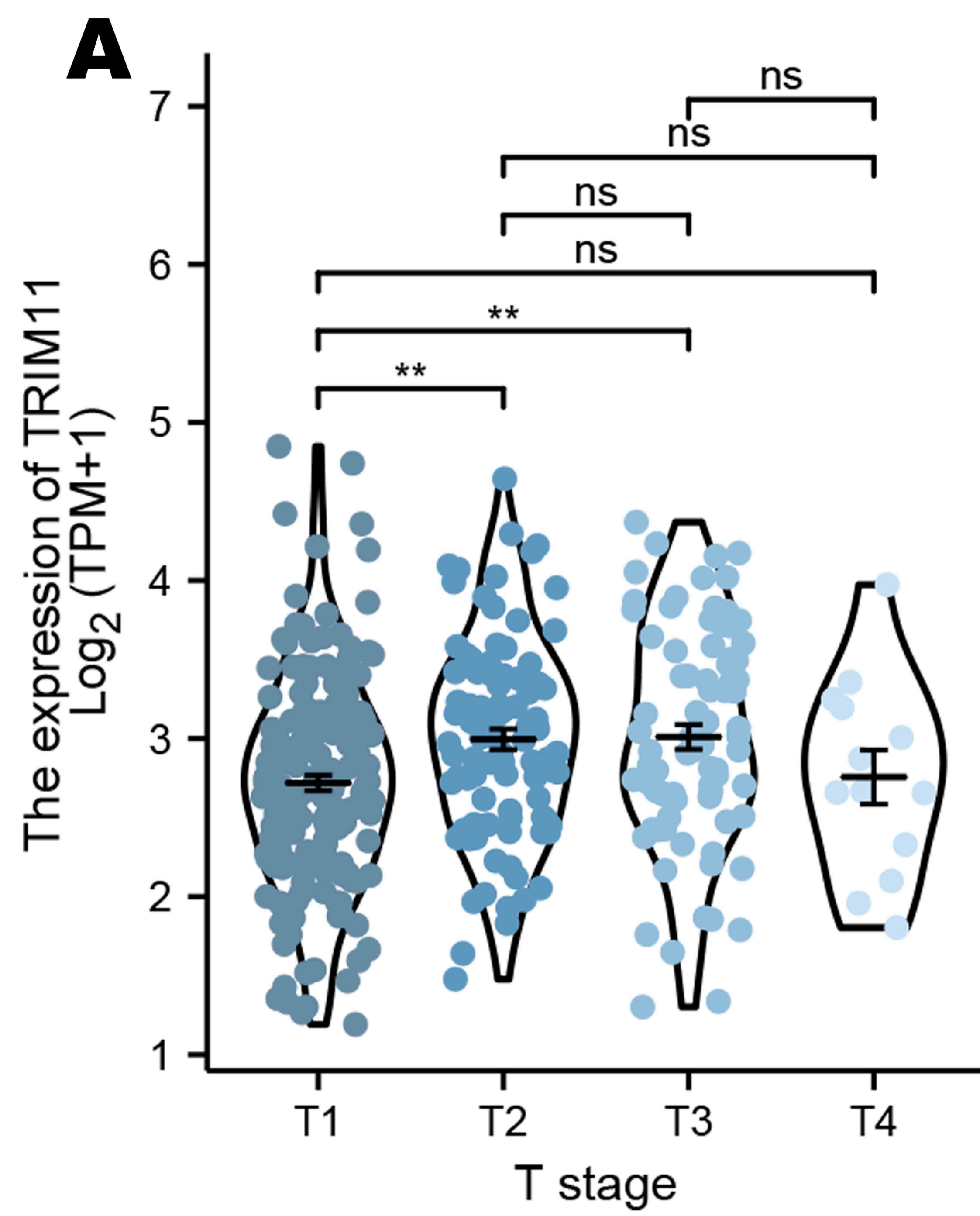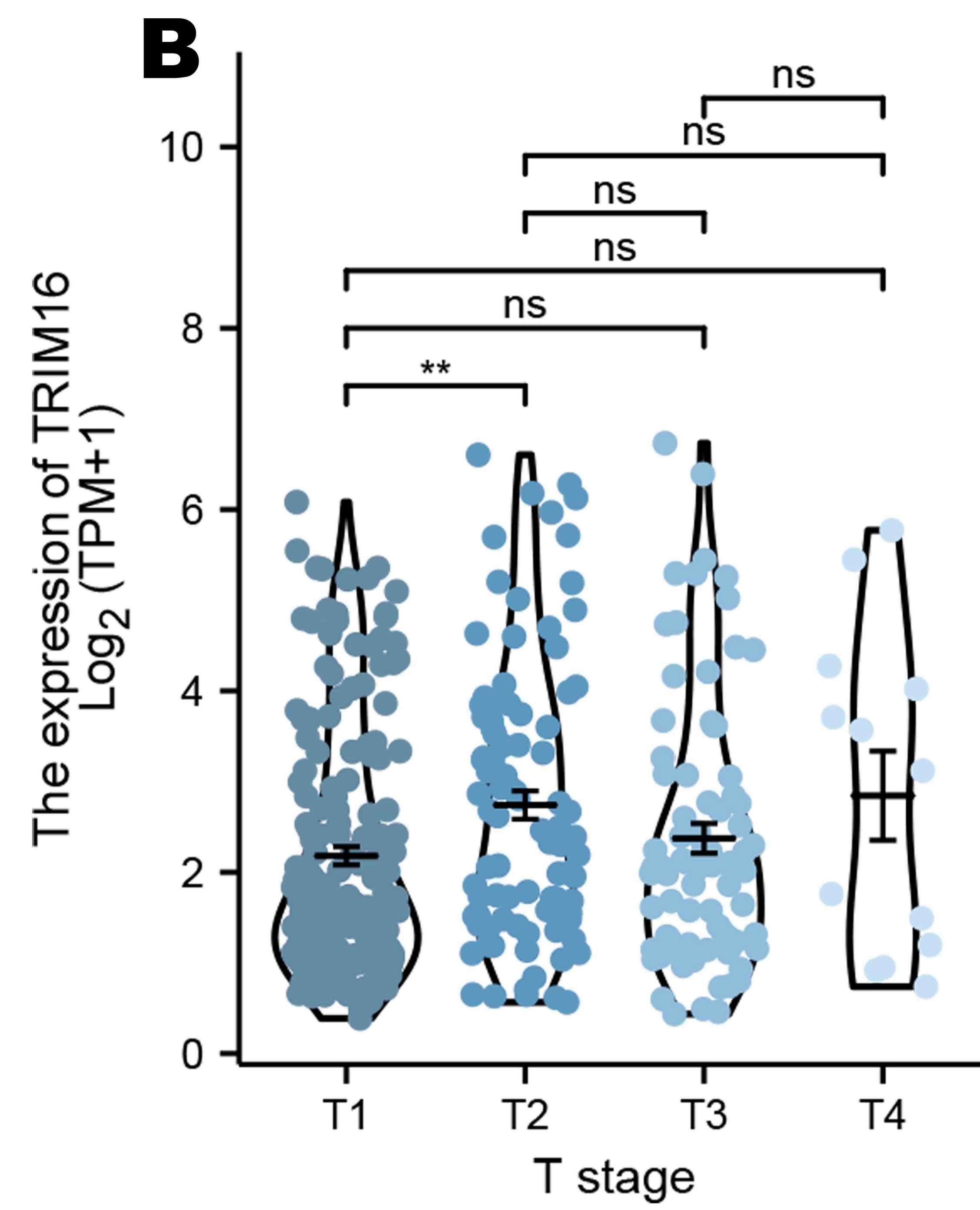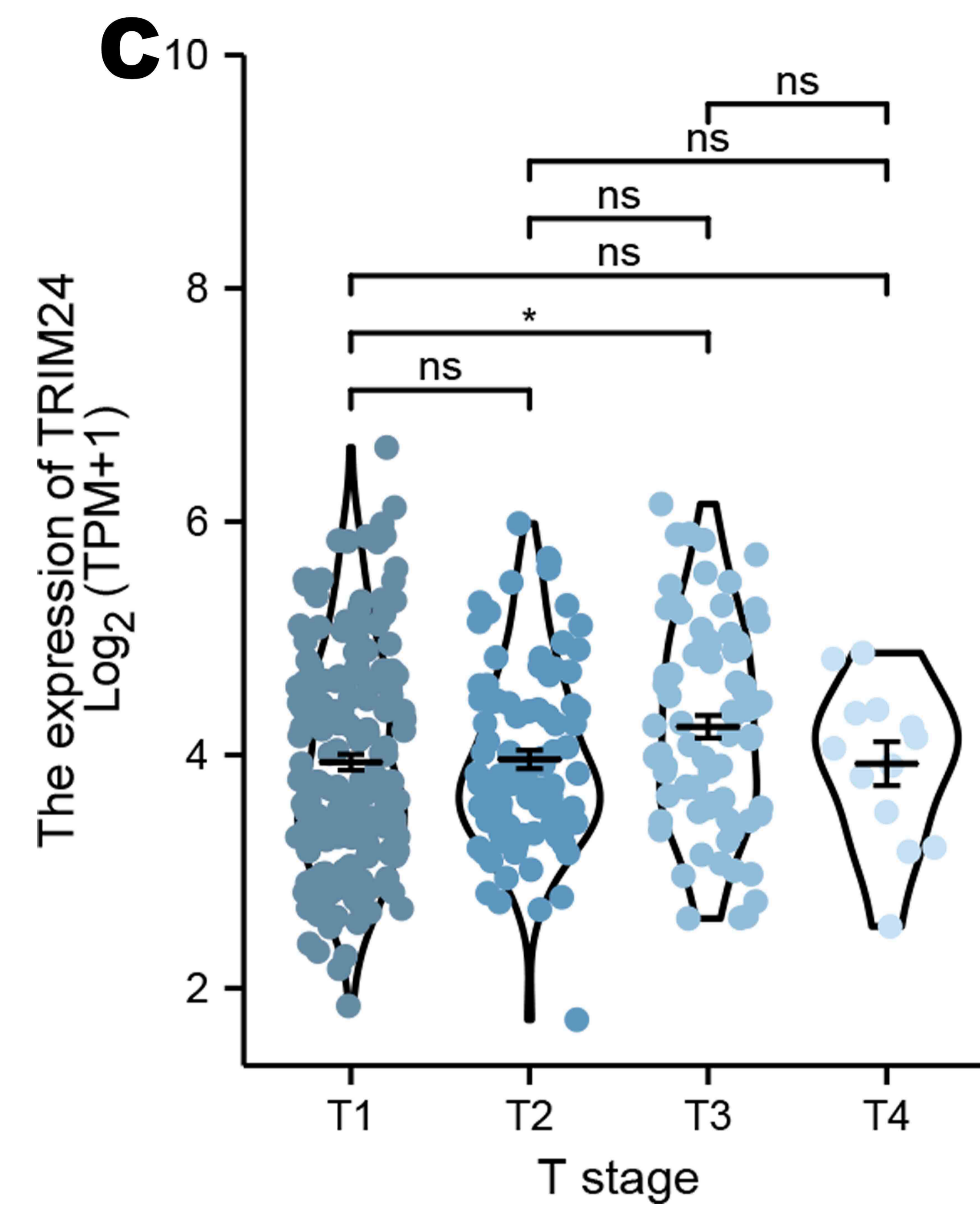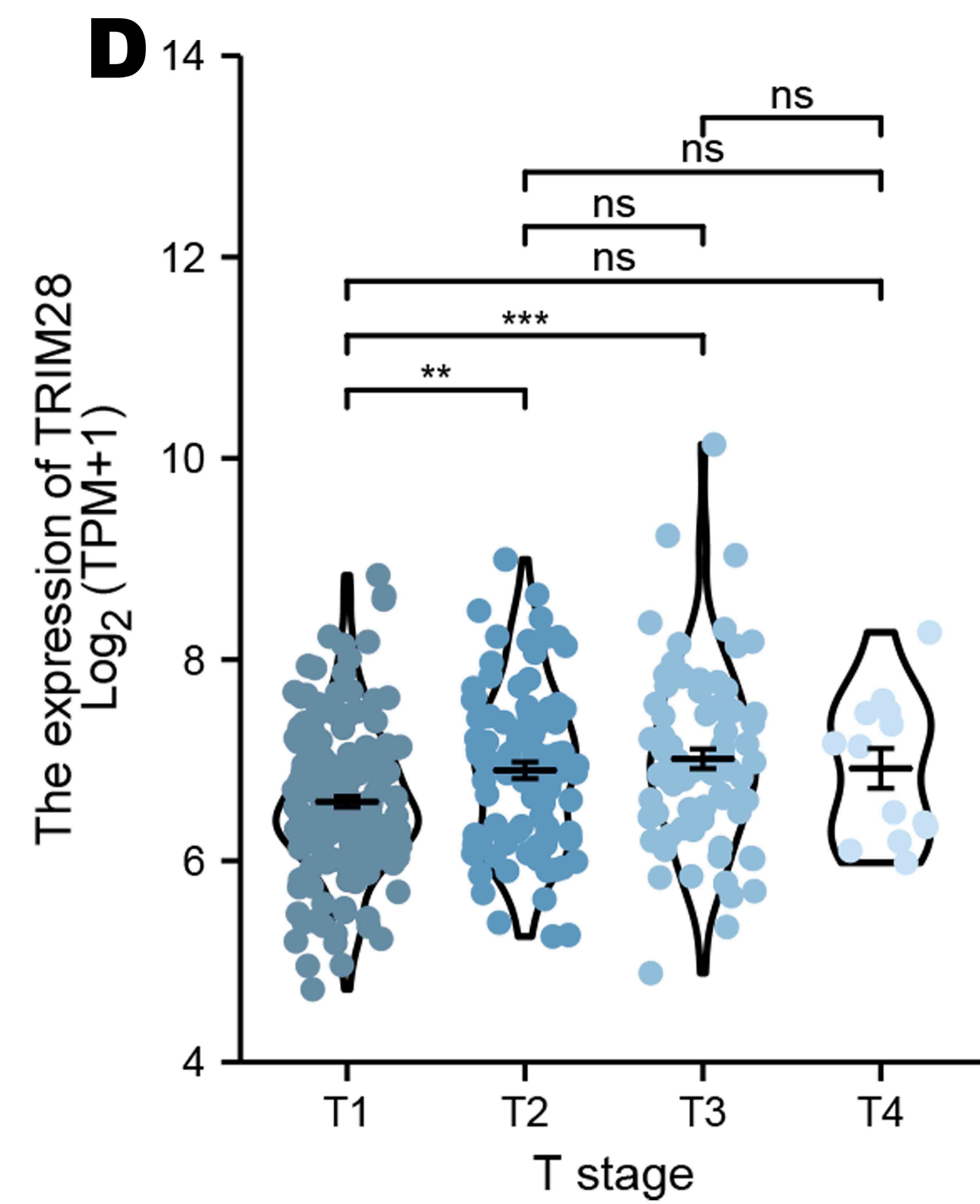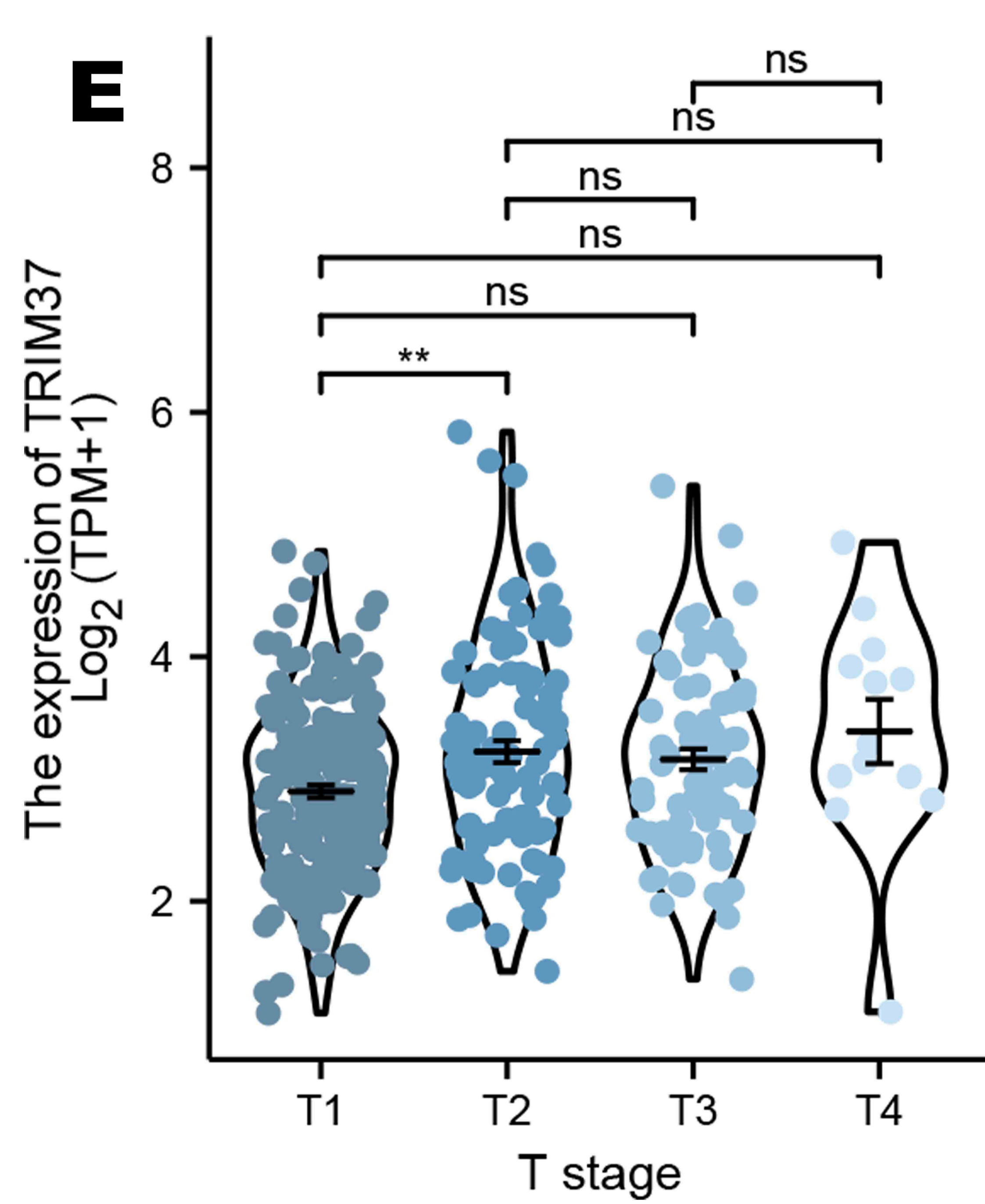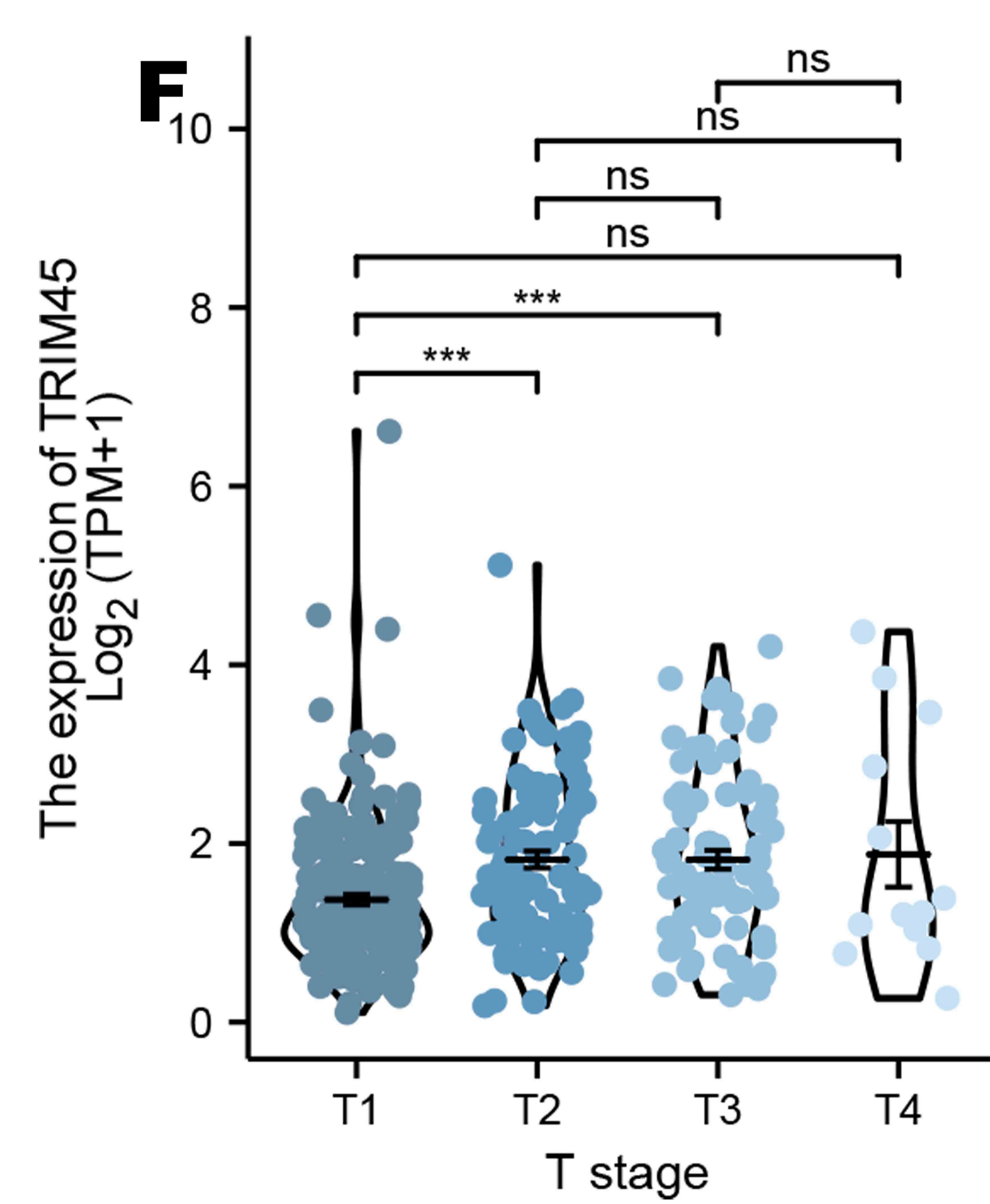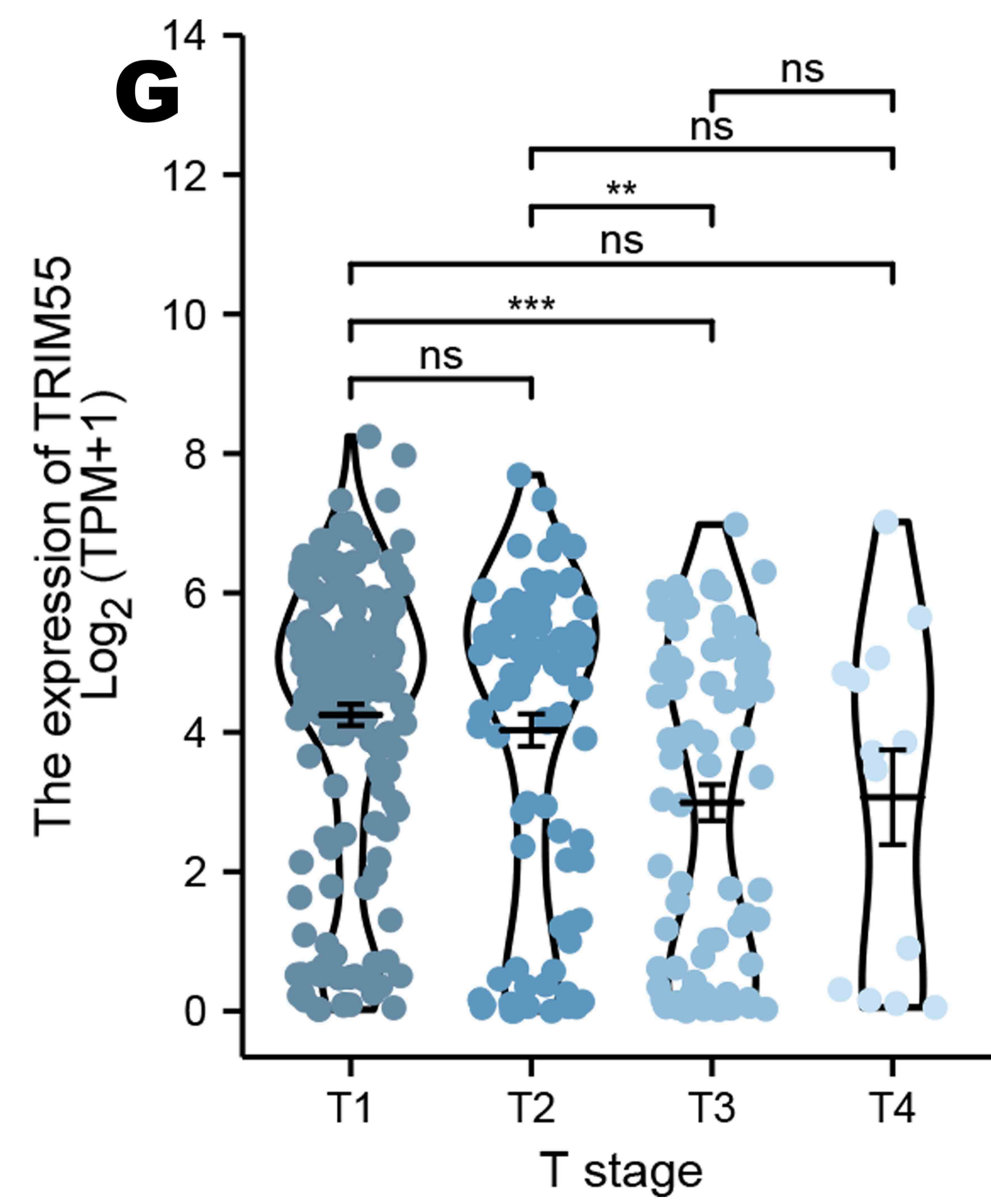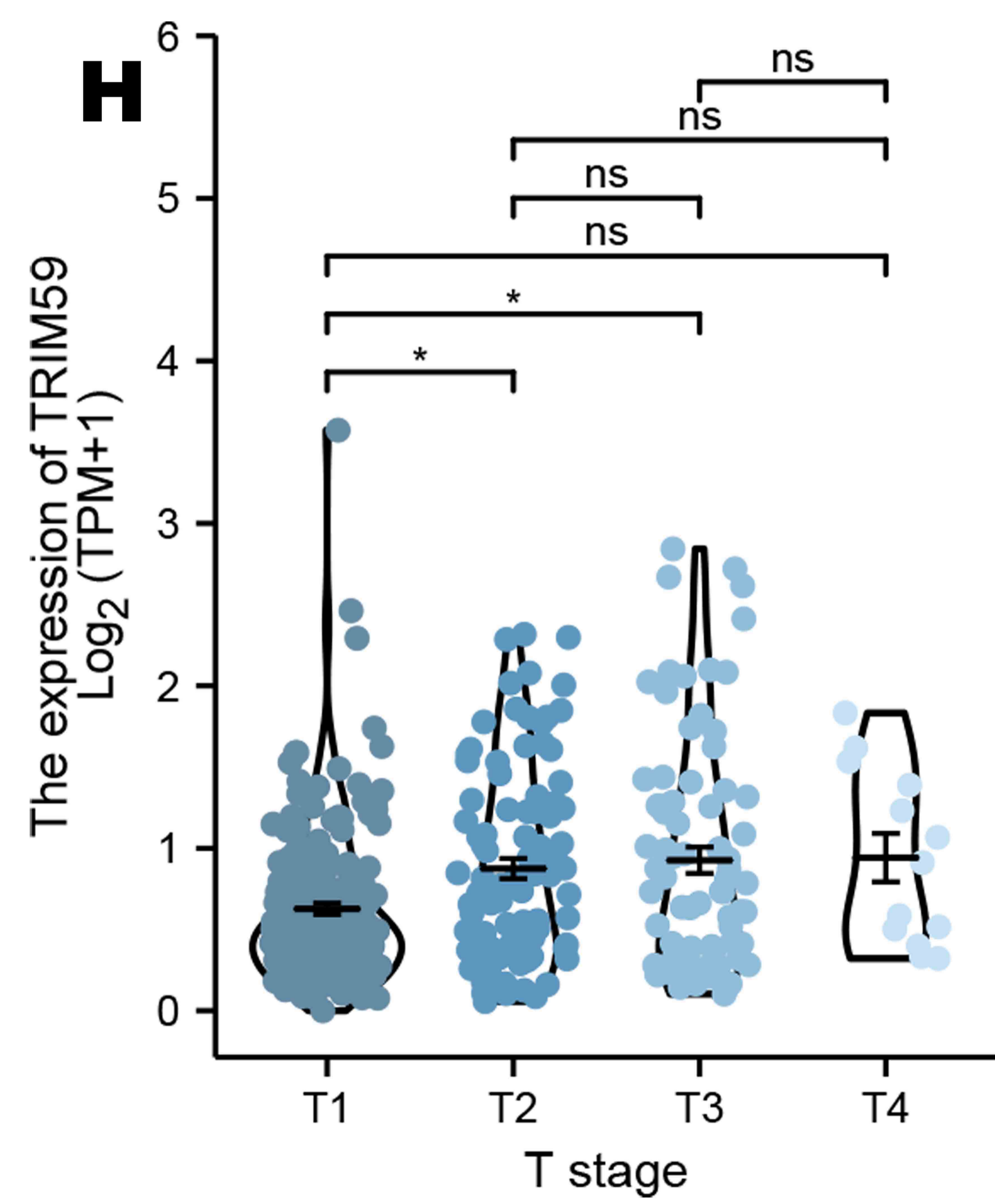

Supplement: Supplementary file 2 — Figure S2 [file CAM4-11-1712-s003.pdf]
