# Supplementary material for: Comprehensive profiling of the TRIpartite motif family to identify pivot genes in hepatocellular carcinoma
Source: Cancer Med. 2022 Feb 9;11(7):1712–31. doi: 10.1002/cam4.4552 (PMC8986146; doi:10.1002/cam4.4552)
Supplement: Supplementary file 3 — Table S1 [file CAM4-11-1712-s005.docx]

| Characteristics | Univariate analysis | |  | Multivariate analysis | |
| --- | --- | --- | --- | --- | --- |
|  | Hazard ratio (95% CI) | P value |  | Hazard ratio (95% CI) | P value |
| T stage (1/2/3/4) | 6.098 (2.879-12.914) | ***<0.001*** |  |  |  |
| N stage (0/1) | 3.612 (0.870-14.991) | 0.077 |  |  |  |
| M stage (0/1) | 5.166 (1.246-21.430) | ***0.024*** |  |  |  |
| TRIM28 (low/high) | 1.538 (0.987-2.396) | 0.057 |  |  |  |
| TRIM37 (low/high) | 1.838 (1.173-2.880) | ***0.008*** |  |  |  |
| TRIM45 (low/high) | 1.532 (0.983-2.389) | 0.060 |  |  |  |
| TRIM59 (low/high) | 1.811 (1.152-2.847) | ***0.010*** |  |  |  |
| Gender (M/F) | 1.230 (0.780-1.937) | 0.373 |  |  |  |
| Age (≤60/>60) | 0.846 (0.543-1.317) | 0.458 |  |  |  |
| Pathologic.stage (I/II/III/IV) | 5.182 (1.259-21.337) | ***0.023*** |  |  |  |
| Child-Pugh.grade (A/B/C) | 3.295 (0.447-24.271) | 0.242 |  |  |  |
| Histologic.grade (G1/2/3/4) | 1.012 (0.247-4.144) | 0.987 |  |  |  |
| Adjacent.hepatic.tissue.inflammation (0/1) | 1.403 (0.768-2.566) | 0.271 |  |  |  |
| AFP(ng/ml) (≤400/>400) | 0.867 (0.450-1.668) | 0.668 |  |  |  |
| Albumin(g/dl) (<3.5/≥3.5) | 1.148 (0.586-2.250) | 0.687 |  |  |  |
| Prothrombin.time (≤4/>4) | 1.778 (1.054-2.999) | ***0.031*** |  | 2.316 (1.013-5.296) | ***0.047*** |
