# Supplementary material for: Comprehensive profiling of the TRIpartite motif family to identify pivot genes in hepatocellular carcinoma
Source: Cancer Med. 2022 Feb 9;11(7):1712–31. doi: 10.1002/cam4.4552 (PMC8986146; doi:10.1002/cam4.4552)
Supplement: Supplementary file 4 — Table S2 [file CAM4-11-1712-s001.docx]

| Characteristics | Univariate analysis | |  | Multivariate analysis | |
| --- | --- | --- | --- | --- | --- |
|  | Hazard ratio (95% CI) | P value |  | Hazard ratio (95% CI) | P value |
| T stage (1/2/3/4) | 2.776 (1.458-5.284) | ***0.002*** |  | 2.533 (1.026-6.253) | ***0.044*** |
| N stage (0/1) | 1.370 (0.338-5.552) | 0.659 |  |  |  |
| M stage (0/1) | 3.476 (1.091-11.076) | ***0.035*** |  |  |  |
| TRIM28 (low/high) | 1.508 (1.127-2.018) | ***0.006*** |  |  |  |
| TRIM37 (low/high) | 1.666 (1.244-2.230) | ***<0.001*** |  | 1.511 (1.011-2.257) | ***0.044*** |
| TRIM45 (low/high) | 1.644 (1.228-2.202) | ***<0.001*** |  |  |  |
| TRIM59 (low/high) | 1.556 (1.161-2.085) | ***0.003*** |  |  |  |
| Gender (M/F) | 1.018 (0.747-1.387) | 0.909 |  |  |  |
| Age (≤60/>60) | 0.960 (0.718-1.284) | 0.783 |  |  |  |
| Pathologic.stage (I/II/III/IV) | 3.519 (1.111-11.150) | ***0.033*** |  |  |  |
| Child-Pugh.grade (A/B/C) | 1.127 (0.157-8.099) | 0.905 |  |  |  |
| Histologic.grade (G1/2/3/4) | 0.911 (0.374-2.220) | 0.838 |  |  |  |
| Adjacent.hepatic.tissue.inflammation (0/1) | 1.238 (0.867-1.768) | 0.241 |  |  |  |
| AFP(ng/ml) (≤400/>400) | 1.045 (0.698-1.563) | 0.832 |  |  |  |
| Albumin(g/dl) (<3.5/≥3.5) | 0.911 (0.618-1.341) | 0.636 |  |  |  |
| Prothrombin.time (≤4/>4) | 1.100 (0.785-1.541) | 0.581 |  |  |  |
