# Supplementary material for: Comprehensive profiling of the TRIpartite motif family to identify pivot genes in hepatocellular carcinoma
Source: Cancer Med. 2022 Feb 9;11(7):1712–31. doi: 10.1002/cam4.4552 (PMC8986146; doi:10.1002/cam4.4552)
Supplement: Supplementary file 5 — Table S3 [file CAM4-11-1712-s006.docx]

| Characteristic  n=187 | level | TRIM28 | | ***p*** | TRIM37 | | ***p*** | TRIM45 | | ***p*** | TRIM59 | | ***p*** |
| --- | --- | --- | --- | --- | --- | --- | --- | --- | --- | --- | --- | --- | --- |
|  |  | Low | High |  | Low | High |  | Low | High |  | Low | High |  |
| T stage n (%) | T1 | 112 (30.2%) | 71 (19.1%) | ***< 0.001*** | 102 (27.5%) | 81 (21.8%) | 0.130 | 114 (30.7%) | 69 (18.6%) | ***< 0.001*** | 108 (29.1%) | 75 (20.2%) | ***0.005*** |
|  | T2 | 38 (10.2%) | 57 (15.4%) |  | 41 (11.1%) | 54 (14.6%) |  | 34 (9.2%) | 61 (16.4%) |  | 39 (10.5%) | 56 (15.1%) |  |
|  | T3 | 29 (7.8%) | 51 (13.7%) |  | 36 (9.7%) | 44 (11.9%) |  | 29 (7.8%) | 51 (13.7%) |  | 32 (8.6%) | 48 (12.9%) |  |
|  | T4 | 6 (1.6%) | 7 (1.9%) |  | 5 (1.3%) | 8 (2.2%) |  | 8 (2.2%) | 5 (1.3%) |  | 5 (1.3%) | 8 (2.2%) |  |
| N stage n (%) | N0 | 119 (46.1%) | 135 (52.3%) | 0.626 | 125 (48.4%) | 129 (50%) | 0.622 | 125 (48.4%) | 129 (50%) | 0.622 | 124 (48.1%) | 130 (50.4%) | 0.623 |
|  | N1 | 1 (0.4%) | 3 (1.2%) |  | 1 (0.4%) | 3 (1.2%) |  | 1 (0.4%) | 3 (1.2%) |  | 1 (0.4%) | 3 (1.2%) |  |
| M stage n (%) | M0 | 125 (46%) | 143 (52.6%) | 1.000 | 135 (49.6%) | 133 (48.9%) | 1.000 | 132 (48.5%) | 136 (50%) | 0.122 | 130 (47.8%) | 138 (50.7%) | 0.361 |
|  | M1 | 2 (0.7%) | 2 (0.7%) |  | 2 (0.7%) | 2 (0.7%) |  | 4 (1.5%) | 0 (0%) |  | 3 (1.1%) | 1 (0.4%) |  |
| Pathologic stage n (%) | I | 104 (29.7%) | 69 (19.7%) | ***< 0.001*** | 97 (27.7%) | 76 (21.7%) | 0.107 | 108 (30.9%) | 65 (18.6%) | ***< 0.001*** | 101 (28.9%) | 72 (20.6%) | ***0.002*** |
|  | II | 36 (10.3%) | 51 (14.6%) |  | 40 (11.4%) | 47 (13.4%) |  | 32 (9.1%) | 55 (15.7%) |  | 38 (10.9%) | 49 (14%) |  |
|  | III | 30 (8.6%) | 55 (15.7%) |  | 35 (10%) | 50 (14.3%) |  | 31 (8.9%) | 54 (15.4%) |  | 31 (8.9%) | 54 (15.4%) |  |
|  | IV | 3 (0.9%) | 2 (0.6%) |  | 3 (0.9%) | 2 (0.6%) |  | 5 (1.4%) | 0 (0%) |  | 4 (1.1%) | 1 (0.3%) |  |
| Tumor status n (%) | Tumor free | 109 (30.7%) | 93 (26.2%) | 0.122 | 112 (31.5%) | 90 (25.4%) | ***0.039*** | 110 (31%) | 92 (25.9%) | 0.078 | 112 (31.5%) | 90 (25.4%) | ***0.021*** |
|  | With tumor | 69 (19.4%) | 84 (23.7%) |  | 67 (18.9%) | 86 (24.2%) |  | 68 (19.2%) | 85 (23.9%) |  | 65 (18.3%) | 88 (24.8%) |  |
| Gender n (%) | F | 58 (15.5%) | 63 (16.8%) | 0.658 | 56 (15%) | 65 (17.4%) | 0.377 | 50 (13.4%) | 71 (19%) | ***0.027*** | 47 (12.6%) | 74 (19.8%) | ***0.004*** |
|  | M | 129 (34.5%) | 124 (33.2%) |  | 131 (35%) | 122 (32.6%) |  | 137 (36.6%) | 116 (31%) |  | 140 (37.4%) | 113 (30.2%) |  |
| BMI n (%) | ≤25 | 77 (22.8%) | 100 (29.7%) | ***0.014*** | 91 (27%) | 86 (25.5%) | 0.881 | 82 (24.3%) | 95 (28.2%) | 0.111 | 82 (24.3%) | 95 (28.2%) | 0.111 |
|  | ＞25 | 92 (27.3%) | 68 (20.2%) |  | 80 (23.7%) | 80 (23.7%) |  | 89 (26.4%) | 71 (21.1%) |  | 89 (26.4%) | 71 (21.1%) |  |
| Histologic grade n (%) | G1 | 36 (9.8%) | 19 (5.1%) | ***< 0.001*** | 33 (8.9%) | 22 (6%) | ***0.030*** | 37 (10%) | 18 (4.9%) | ***< 0.001*** | 39 (10.6%) | 16 (4.3%) | ***< 0.001*** |
|  | G2 | 102 (27.6%) | 76 (20.6%) |  | 96 (26%) | 82 (22.2%) |  | 99 (26.8%) | 79 (21.4%) |  | 98 (26.6%) | 80 (21.7%) |  |
|  | G3 | 44 (11.9%) | 80 (21.7%) |  | 53 (14.4%) | 71 (19.2%) |  | 46 (12.5%) | 78 (21.1%) |  | 44 (11.9%) | 80 (21.7%) |  |
|  | G4 | 4 (1.1%) | 8 (2.2%) |  | 3 (0.8%) | 9 (2.4%) |  | 4 (1.1%) | 8 (2.2%) |  | 4 (1.1%) | 8 (2.2%) |  |
| AFP(ng/ml) n (%) | ≤400 | 137 (48.9%) | 78 (27.9%) | ***< 0.001*** | 117 (41.8%) | 98 (35%) | 0.553 | 118 (42.1%) | 97 (34.6%) | 0.050 | 128 (45.7%) | 87 (31.1%) | ***< 0.001*** |
|  | ＞400 | 12 (4.3%) | 53 (18.9%) |  | 32 (11.4%) | 33 (11.8%) |  | 26 (9.3%) | 39 (13.9%) |  | 17 (6.1%) | 48 (17.1%) |  |
| Child-Pugh grade n (%) | A | 122 (50.6%) | 97 (40.2%) | 0.643 | 123 (51%) | 96 (39.8%) | 0.304 | 115 (47.7%) | 104 (43.2%) | 0.904 | 125 (51.9%) | 94 (39%) | 0.895 |
|  | B | 10 (4.1%) | 11 (4.6%) |  | 9 (3.7%) | 12 (5%) |  | 12 (5%) | 9 (3.7%) |  | 13 (5.4%) | 8 (3.3%) |  |
|  | C | 1 (0.4%) | 0 (0%) |  | 1 (0.4%) | 0 (0%) |  | 1 (0.4%) | 0 (0%) |  | 1 (0.4%) | 0 (0%) |  |
| Vascular invasion n (%) | No | 117 (36.8%) | 91 (28.6%) | 0.159 | 115 (36.2%) | 93 (29.2%) | 0.531 | 121 (38.1%) | 87 (27.4%) | ***0.012*** | 116 (36.5%) | 92 (28.9%) | 0.139 |
|  | Yes | 52 (16.4%) | 58 (18.2%) |  | 56 (17.6%) | 54 (17%) |  | 47 (14.8%) | 63 (19.8%) |  | 51 (16%) | 59 (18.6%) |  |
| Fibrosis score n (%) | 0 | 47 (21.9%) | 28 (13%) | 0.188 | 38 (17.7%) | 37 (17.2%) | 0.622 | 45 (20.9%) | 30 (14%) | 0.643 | 47 (21.9%) | 28 (13%) | 0.429 |
|  | 1/2 | 16 (7.4%) | 15 (7%) |  | 20 (9.3%) | 11 (5.1%) |  | 15 (7%) | 16 (7.4%) |  | 16 (7.4%) | 15 (7%) |  |
|  | 3/4 | 11 (5.1%) | 17 (7.9%) |  | 16 (7.4%) | 12 (5.6%) |  | 14 (6.5%) | 14 (6.5%) |  | 13 (6%) | 15 (7%) |  |
|  | 5/6 | 46 (21.4%) | 35 (16.3%) |  | 44 (20.5%) | 37 (17.2%) |  | 43 (20%) | 38 (17.7%) |  | 48 (22.3%) | 33 (15.3%) |  |
| OS event n (%) | Alive | 131 (35%) | 113 (30.2%) | 0.065 | 133 (35.6%) | 111 (29.7%) | ***0.023*** | 128 (34.2%) | 116 (31%) | 0.232 | 132 (35.3%) | 112 (29.9%) | ***0.039*** |
|  | Dead | 56 (15%) | 74 (19.8%) |  | 54 (14.4%) | 76 (20.3%) |  | 59 (15.8%) | 71 (19%) |  | 55 (14.7%) | 75 (20.1%) |  |
